# De-identifying a public use microdata file from the Canadian national discharge abstract database

**DOI:** 10.1186/1472-6947-11-53

**Published:** 2011-08-23

**Authors:** Khaled El Emam, David Paton, Fida Dankar, Gunes Koru

**Affiliations:** 1 CHEO Research Institute, 401 Smyth Road, Ottawa, ON, Canada, K1H 8L1; 2 Department of Paediatrics, University of Ottawa, 401 Smyth Road, Ottawa, ON, Canada, K1H 8L1; 3 Canadian Institute for Health Information, 495 Richmond Road, Suite 600 Ottawa, ON, Canada, K2A 4H6; 4 Information Systems, University of Maryland at Baltimore County, Baltimore, USA

## Abstract

**Abstract:**

**Background**

The Canadian Institute for Health Information (CIHI) collects hospital discharge abstract data (DAD) from Canadian provinces and territories. There are many demands for the disclosure of this data for research and analysis to inform policy making. To expedite the disclosure of data for some of these purposes, the construction of a DAD public use microdata file (PUMF) was considered. Such purposes include: confirming some published results, providing broader feedback to CIHI to improve data quality, training students and fellows, providing an easily accessible data set for researchers to prepare for analyses on the full DAD data set, and serve as a large health data set for computer scientists and statisticians to evaluate analysis and data mining techniques. The objective of this study was to measure the probability of re-identification for records in a PUMF, and to de-identify a national DAD PUMF consisting of 10% of records.

**Methods:**

Plausible attacks on a PUMF were evaluated. Based on these attacks, the 2008-2009 national DAD was de-identified. A new algorithm was developed to minimize the amount of suppression while maximizing the precision of the data. The acceptable threshold for the probability of correct re-identification of a record was set at between 0.04 and 0.05. Information loss was measured in terms of the extent of suppression and entropy.

**Results:**

Two different PUMF files were produced, one with geographic information, and one with no geographic information but more clinical information. At a threshold of 0.05, the maximum proportion of records with the diagnosis code suppressed was 20%, but these suppressions represented only 8-9% of all values in the DAD. Our suppression algorithm has less information loss than a more traditional approach to suppression. Smaller regions, patients with longer stays, and age groups that are infrequently admitted to hospitals tend to be the ones with the highest rates of suppression.

**Conclusions:**

The strategies we used to maximize data utility and minimize information loss can result in a PUMF that would be useful for the specific purposes noted earlier. However, to create a more detailed file with less information loss suitable for more complex health services research, the risk would need to be mitigated by requiring the data recipient to commit to a data sharing agreement.

## Background

There are increasing pressures to make raw individual-level data more readily available for research and policy making purposes [1-5]. This should be pursued as there are many benefits to doing so [1-15]. National statistical agencies have responded to such demands by creating public use microdata files (PUMFs) [16]. For instance, PUMFs from census data and population surveys are often created [17-22], and recently the Centers for Medicare and Medicaid Services has started work on creating 5% PUMFs from claims databases (e.g., inpatient and outpatient claims) [23,24].

Making data available as a PUMF entails disclosing individual level data with minimal restrictions or conditions on access. The ideal PUMF provides as much detail as possible short of disclosing raw files where the patients are readily identifiable [15].

The focus of this paper is on the creation of a PUMF for the Canadian national discharge abstract database (DAD).

In the US, 48 states collect data on inpatients [25], and 26 states make their DADs available through the Agency for Healthcare Research and Quality (AHRQ) [26]. This data can be purchased for the purposes of research or other approved use. All purchasers must also sign a data use agreement prohibiting the re-identification of individuals in the data set. The equivalent agency in Canada is the Canadian Institute for Health Information (CIHI), which collects DAD data from hospitals across the country [27] and makes it available through an application process, as well as performing its own analyses on the data. Data disclosed by CIHI are somewhat de-identified (it contains no direct identifiers), and are only provided when there is a data sharing agreement [28].

Discharge abstract data has been used for a diverse set of research and analysis purposes, including public safety and injury surveillance and prevention, public health, disease surveillance, public reporting for informed purchasing and comparative reports, quality assessment and performance improvement, and health services research [29].

CIHI was considering the creation of a public use microdata file (PUMF) for the national DAD. The availability of a DAD PUMF would make it easier for the research community to confirm some published results, provide broader feedback to CIHI to improve data quality, can be a tool for training students and fellows, provide an easily accessible data set for researchers to prepare for analyses on the full DAD data set, and serve as a large data set for computer scientists and statisticians to evaluate analysis and data mining techniques. A PUMF would be readily available with no application nor waiting time, at no cost to the requestor, and it would not impose an on-going burden on the organization in terms of processing requests for data. It would complement the existing method of providing data through the regular data application process.

Two primary concerns with the creation of a PUMF were to ensure that the probability of re-identification of the patients is acceptably low and that the disclosed PUMF had sufficient utility to end-users.

The purpose of our study was to create a prototype PUMF. The outcome of this analysis was intended to inform the decision on whether to proceed with an actual PUMF. A critical criterion for making that decision was whether the resulting PUMF still had utility for end-users.

The contributions of this study are: (a) an analysis of plausible re-identification attacks on a Canadian DAD PUMF, (b) a set of new re-identification metrics were developed for evaluating these attacks, (c) a new set of strategies for maximizing data utility when de-identifying data were formulated, (d) a new efficient algorithm for the suppression of large data sets was developed, and (e) we present the results evaluating the probability of re-identification and the de-identification of a Canadian national DAD PUMF.

## Definitions

Here we provide some basic definitions that we use throughout the paper, review related work for the de-identification of individual-level data (also known as microdata), and present metrics for evaluating information loss due to de-identification.

### Categories of Variables

It is useful to differentiate among the different types of variables in a data set. The way the variables are handled during the de-identification process will depend on how they are categorized. We make a distinction among four types of variables [30,31], and these are illustrated in Table 1:

**Table 1:** Example data

	IDENTIFYING VARIABLE		QUASI-IDENTIFIERS		SENSITIVE VARIABLES		Other Variables
**ID**	**Name**	**Telephone Number**	**Sex**	**Year of Birth**	**Lab Test**	**Lab Result**	**PayDelay**

1	John Smith	(412) 688-5468	Male	1959	Albumin, Serum	4.8	37

2	Alan Smith	(413) 822-5074	Male	1969	Creatine kinase	86	36

3	Alice Brown	(416) 886-5314	Female	1955	Alkaline Phosphatase	66	52

4	Hercules Green	(613) 763-5254	Male	1959	Bilirubin	Negative	36

5	Alicia Freds	(613) 586-6222	Female	1942	BUN/Creatinine Ratio	17	82

6	Gill Stringer	(954) 699-5423	Female	1975	Calcium, Serum	9.2	34

7	Marie Kirkpatrick	(416) 786-6212	Female	1966	Free Thyroxine Index	2.7	23

8	Leslie Hall	(905) 668-6581	Female	1987	Globulin, Total	3.5	9

9	Douglas Henry	(416) 423-5965	Male	1959	B-type natriuretic peptide	134.1	38

10	Fred Thompson	(416) 421-7719	Male	1967	Creatine kinase	80	21

11	Joe Doe	(705) 727-7808	Male	1968	Alanine aminotransferase	24	33

12	Lillian Barley	(416) 695-4669	Female	1955	Cancer antigen 125	86	28

13	Deitmar Plank	(416) 603-5526	Male	1967	Creatine kinase	327	37

14	Anderson Hoyt	(905) 388-2851	Male	1967	Creatine kinase	82	16

15	Alexandra Knight	(416) 539-4200	Female	1966	Creatinine	0.78	44

16	Helene Arnold	(519) 631-0587	Female	1955	Triglycerides	147	59

17	Almond Zipf	(519) 515-8500	Male	1967	Creatine kinase	73	20

18	Britney Goldman	(613) 737-7870	Female	1956	Monocytes	12	34

19	Lisa Marie	(902) 473-2383	Female	1956	HDL Cholesterol	68	141

20	William Cooper	(905) 763-6852	Male	1978	Neutrophils	83	21

21	Kathy Last	(705) 424-1266	Female	1966	Prothrombin Time	16.9	23

22	Deitmar Plank	(519) 831-2330	Male	1967	Creatine kinase	68	16

23	Anderson Hoyt	(705) 652-6215	Male	1971	White Blood Cell Count	13.0	151

24	Alexandra Knight	(416) 813-5873	Female	1954	Hemoglobin	14.8	34

25	Helene Arnold	(705) 663-1801	Female	1977	Lipase, Serum	37	27

26	Anderson Heft	(416) 813-6498	Male	1944	Cholesterol, Total	147	18

27	Almond Zipf	(617) 667-9540	Male	1965	Hematocrit	45.3	53

This hypothetical example table is used to illustrate a number of concepts that we use throughout the paper.

#### Directly Identifying variables

One or more direct identifiers can be used to uniquely identify an individual, either by themselves or in combination with other readily available information. For example, there are more than 200 people named "John Smith" in Ontario, therefore the name by itself would not be directly identifying, but in combination with the address it would be directly identifying information. A telephone number is not directly identifying by itself, but in combination with the readily available White Pages it becomes so. Other examples of directly identifying variables include email address, health insurance card number, credit card number, and social insurance number. These numbers are identifying because there exist public and/or private databases that an adversary can plausibly get access to where these numbers can lead directly, and uniquely, to an identity. For example, Table 1 shows the names and telephone numbers of individuals. In that case the name and number would be considered as identifying variables.

#### Indirectly identifying variables (quasi-identifiers)

The quasi-identifiers are the background knowledge variables about individuals in the DAD that an adversary can use, individually or in combination, to probabilistically re-identify a record. If an adversary does not have background knowledge of a variable then it cannot be a quasi-identifier. The manner in which an adversary can obtain such background knowledge will determine which attacks on a data set are plausible. For example, the background knowledge may be available because the adversary knows a particular target individual in the disclosed data set, an individual in the data set has a visible characteristic that is also described in the data set, or the background knowledge exists in a public or semi-pubic registry. Examples of quasi-identifiers include sex, date of birth or age, locations (such as postal codes, census geography, information about proximity to known or unique landmarks), language spoken at home, ethnic origin, aboriginal identity, total years of schooling, marital status, criminal history, total income, visible minority status, activity difficulties/reductions, profession, event dates (such as admission, discharge, procedure, death, specimen collection, visit/encounter), codes (such as diagnosis codes, procedure codes, and adverse event codes), country of birth, birth weight, and birth plurality.

For example, Table 1 shows the patient sex and year of birth (from which an age can be derived) as quasi-identifiers.

#### Sensitive variables

These are the variables that are not really useful for determining an individual's identity but contain sensitive health information about the individuals. Examples of sensitive variables are laboratory test results and drug dosage information. In Table 1 the lab test that was ordered and the test results are the sensitive variables.

#### Other variables

Any variable in the data set which does not fall into one of the above categories falls into this 'catch all' category. For example, in Table 1 we see the variable PayDelay, which indicates how long (in days) it took the insurer to pay the provider. In general, this information is not considered sensitive and would be quite difficult for an adversary to use for re-identification attack purposes.

Sometimes a variable classified as a quasi-identifier in one context is classified as a sensitive variable in another context. This will depend on the assumptions made about the adversary's background knowledge, and these assumptions will be situation dependent.

Individuals can be re-identified because of the directly identifying variables and the quasi-identifiers. There are no directly identifying variables in the proposed DAD PUMF. Therefore, the critical question is which variables are quasi-identifiers. The specific quasi-identifiers in the DAD are discussed below.

### Equivalence Classes

All the records that share the same values on a set of quasi-identifiers are called an *equivalence class*. For example, consider the quasi-identifiers in Table 1, sex and age. All the records in Table 1 about males born in 1967 are an equivalence class (these have an ID of 10, 13, 14, 17, and 22). Equivalence class sizes for a data concept (such as age) potentially change during de-identification. For example, there may be 5 records for males born in 1967. When the precision of age is reduced to a five year interval, then there are 8 records for males born between 1965 and 1969 (these have an ID of 2, 10, 11, 13, 14, 17, 22, and 27). In general there is a trade off between the level of detail provided for a data concept and the size of the corresponding equivalence classes, with more detail associated with smaller equivalence classes.

### Types of Disclosure

There are two kinds of disclosure that are of concern: identity disclosure and attribute disclosure [32,33]. The first is when an adversary can assign an identity to a record in the PUMF. For example, if the adversary would be able to determine that record number 3 belongs to patient Alice Brown in Table 1 using only the quasi-identifiers, then this is identity disclosure. The second type of disclosure is when an adversary learns a sensitive attribute about a patient in the database with a sufficiently high probability without knowing which specific record belongs to that patient [32,34]. For example, in Table 1 all males born in 1967 had a creatine kinease lab test. Assume that an adversary does not need to know which record belongs to Almond Zipf (record ID 17). Since Almond is male and was born in 1967 then the adversary will discover something new about him (that he had a test often given to individuals showing symptoms of a heart attack). This is attribute disclosure.

Known re-identifications of personal information that have actually occurred are identity disclosures, for example: (a) reporters re-identified an individual's records from web search queries publicly posted by AOL [35-37], (b) students re-identified individuals in the Chicago homicide database by linking it with the social security death index [38], (c) at least one individual was believed to be re-identified by linking their movie ratings in a publicly disclosed Netflix file to another public movie ratings database [39], (d) the insurance claims records of the governor of Massachusetts were re-identified by linking a claims database sold by the state employees' insurer with the voter registration list [31], (e) an expert witness re-identified most of the records in a neuroblastoma registry [40,41], (f) a national broadcaster matched the adverse drug event database with public obituaries to re-identify a 26 year old girl who died while taking a drug and did a documentary on the drug afterwards [42], (g) an individual in a prescriptions record database was re-identified by a neighbour [43], and (h) the Department of Health and Human Services in the US linked a large medical database with a commercial database and re-identified a number of individuals [44].

Therefore in the current analysis we only focused on identity disclosure. This does not mean that attribute disclosure is not important to consider (i.e., absence of evidence does not mean evidence of absence). However, in terms of focus, we address only identity disclosure in the current study.

## Disclosure Control for Microdata

Statistical and computational disclosure control methods can be applied by a data custodian to protect against identity disclosure [45,46]. Disclosure control methods are concerned with both microdata [30,47-53] and tabular data [49,54-58]. The term "microdata" means individual-level data. Tabular data may contain frequencies or aggregate statistics (e.g., means and standard deviations) in the table's cells. Given that the DAD PUMF is a microdata disclosure, we are only concerned with methods for the de-identification of microdata.

De-identification methods for microdata can be broadly categorized as perturbative and non-perturbative [59]. The perturbative methods distort the truthfulness of the records in a data set. However, perturbation can be performed by preserving the aggregated properties of the data. Many perturbation methods have been explored, and some of these are summarized below.

*Random noise *can be added to continuous data without changing the correlation structure of the original data [60-63]. Gaussian noise is frequently used for this purpose. However, there are circumstances where noise can be filtered out of the data to recover the original information [64]. Another approach for both continuous and categorical data is to apply perturbations by considering the *underlying probability distribution *[65]: first, the underlying distribution is discovered, then distorted series are produced and the original series are replaced with the distorted series. *Microaggregation *[66-71] can be another alternative for perturbation, which creates small clusters of data and uses an aggregated value (e.g., average or median) instead of actual values. Some guidance exists on the appropriate size of these clusters [69]. *Re-sampling *[72] is a random perturbation method which resamples individual values in the data set [59]. Another approach is *lossy compression *which treats the continuous data as an image and applies a compression such as JPEG (Joint Photographic Experts Group 2001) [73,74]. *Multiple imputation *utilizes techniques developed to deal with missing data [75]. For example, for each continuous variable, a randomized regression can be used to estimate a replacement value [76]. *Vector camouflage *is another perturbative method which provides unlimited and correct interval answers to database queries without revealing original values [77]. The *Post-Randomization Method *(*PRAM*) is a probabilistic method used for categorical data [78]. A Markov matrix is used to change the scores assigned to categorical variables based on previously determined probabilities. PRAM can be thought of as a general approach comprising other techniques such as adding noise, suppressing data, and recoding data. *Rank swapping *is perturbation method which swaps the values of a variable within a specified range of ranks. It can be applied to ordinal or numerical variables. *Rounding *methods perturb by rounding values to a value in a determined rounding set [49].

The non-perturbative techniques preserve the truthfulness of data. *Subsampling *techniques only publish a subset of the records in a data set [79]. This can be more useful for categorical data compared to numerical data because if an adversary performs a matching attack using an external data set, continuous values are more likely to be unique. *Global recoding *is a non-perturbative technique that applies to all records in a data set. Continuous variables can be grouped into discretized values representing bins; for categorical variables, the variable values can be grouped into larger categories. In certain cases, top or bottom values of a variable can be grouped and published as one value known as *top/bottom recoding*, respectively. The statistical disclosure tool mu-argus can apply global recoding [80]. *Suppression *can be also used to either eliminate some values that cannot be published (e.g., stigmatized diseases) or outlier values that increase the re-identification probability.

## The k-Anonymity Criterion

A popular de-identification criterion is *k-anonymity *[81-86]. With k-anonymity an original data set containing personal health information can be transformed to protect against identity disclosure. A k-anonymized data set has the property that each record is similar to at least another k-1 other records on the quasi-identifiers. For example, if k = 5 and the quasi-identifiers are age and sex, then a k-anonymized data set has at least 5 records for each value combination of age and gender.

Various methods can be used to satisfy the k-anonymity criterion. For example, some authors have used micro-aggregation [52]. However, when dealing with health data, non-perturbative methods are favoured because they preserve the truthfulness of data [87]. Furthermore, some of the most common implementations of k-anonymity use non-perturbative techniques such as global recoding and suppression [81-86]. The data analysts we consulted with were more comfortable with global recoding and suppression because their impact on data analysis was clearer to them. This is an important factor because we wanted to ensure the acceptability of the PUMF among data analysts. For this reason, our study uses global recoding, a form of generalization, and suppression.

After the quasi-identifiers are generalized the equivalence class sizes would be computed. For any equivalence class that is smaller than k, suppression is applied. Therefore, generalization by itself will not achieve k-anonymity, and suppression is only applied to the small equivalence classes.

## Measuring Information Loss

During de-identification, the other side of the coin is data utility. A trade-off exists between privacy and utility. If an optimal trade-off can be found, patient privacy can be preserved while data users are satisfied with the data utility. The data utility can be measured by information loss metrics. Typically, lower levels of information loss are associated with higher data utility, and vice versa.

The extent of suppression performed to the data is an important indicator of information loss. Although the extent of suppression has known disadvantages as an information loss metric [87], it provides an intuitive way for an expert data analyst to gauge data quality. The more suppressed records and/or individual values in a data set the greater the potential biases introduced in an analysis of the data.

A reasonable quantitative assessment of information loss could be based on comparing the analysis results obtained from the original and disclosed (de-identified) data [88]. However, this is difficult to achieve because the potential uses of data can vary and it is difficult to predict all of them in advance. In the case of the DAD PUMF it is not possible to know with precision a priori all the ways that data recipients can analyze that data, which can include statistical as well as machine learning methods. In fact, one purpose of creating a PUMF is to encourage the development of novel data modeling and data mining techniques.

Despite the lack of universally acceptable information loss criteria or metrics, it has been argued that there is little information loss if a data set is *valid and interesting *[89]. A de-identified data set is considered *valid *if it preserves (i) means and co-variances in a small subset of records, (ii) marginal values in a few tabulations of the data, and (iii) at least one distributional characteristic. A data set is called *interesting*, if six variables on important subsets of records can be validly analyzed. While a useful starting point, this definition can only be meaningfully operationalized if there is some knowledge of the analysis that will be performed on the de-identified data. Another suggested approach is to examine the function that maps original records to the protected records [88]. As this function gets closer to the identity function, the information loss will decrease, and vice versa.

Information loss metrics for continuous data include comparing the original and de-identified data sets on the mean square error, mean absolute error, and mean variation [59,90]. Such metrics cannot be easily computed for categorical variables; therefore, three methods are suggested [59,88]: (i) direct comparisons based on a distance definition using category ranges, (ii) comparison of contingency tables, and (iii) entropy-based measures.

An entropy [91,92] metric was used in a number of studies to measure information loss [49,93,94] where suppression, global recoding, and PRAM were used. Recently, the entropy metrics described in [49,93] were extended to deal with the non-uniform distributions, and the resulting measure has been called non-uniform entropy [95] and has been used specifically in k-anonymity algorithms [96].

Samarati used *height *as an information loss metric [81]. Height indicates the generalization level in a quasi-identifier generalization hierarchy. Greater height means more information loss. Height is considered a weaker metric compared to non-uniform entropy because it does not take into account the information loss contributed by individual variables [87].

Another information loss metric based on the generalization hierarchy is Precision or *Prec *[83,97]. For every variable, the ratio of the number of generalization steps applied to the total number of possible generalization steps (total height of the generalization hierarchy) gives the amount of information loss for that particular variable. Overall, *Prec *information loss is the average of the *Prec *values across all quasi-identifiers in the data set.

A frequently used information loss metric is the Discernability Metric [98-105]. The discernability metric (*DM*) assigns a penalty to every record that is proportional to the number of records that are indistinguishable from it. DM has been used often in the computational disclosure control literature.

The *minimal distortion *(*MD*) metric measures the dissimilarities between the original and de-identified records [30,31,106]. This charges a unit of penalty to each generalized or suppressed instance of a value. While both *DM *and *MD *can assess the level of distortion, *DM *has an advantage over *MD *in the sense that *DM *can differentiate how much indistinguishability increased by going from the original to de-identified data set [107].

Information loss caused by generalizations can also measured by using the *ILoss *metric [108]. This metric captures the fraction of domain values generalized to a certain value [45]. *ILoss *for a record is calculated by finding the sum of the *ILoss *values over all variable values. Different weights can be applied to different variables while obtaining this sum. Similarly, the overall *ILoss *for a data set can be obtained by adding up the *ILoss *values found for the records.

Iyengar [109] used a classification metric, CM, which assigns a penalty to a record if suppressions or generalizations assigns the record to a different majority class. This metric is applied to the training set and requires a classification method to be used. The associated problem is that the exact classification approach needed may not be known at the time of data publishing. Fung et al. [110,111] used a metric called *IGPL *to measure a trade-off between information gain (*IG*) and privacy loss (*PL*). *IGPL *is obtained by dividing *IG *by *PL *incremented by 1. The formulas for *IG *and *PL *can be seen in [45].

## Methods

In this section we explain how the Canadian DAD PUMF was de-identified and describe the evaluations that were performed on it.

### PUMF Specifications

Our analysis used the 2008-2009 DAD. Only acute care inpatient cases were included. The DAD excluded hospitalizations in Quebec. Cases with diagnoses or interventions that indicated abortion or HIV were excluded. These were removed because they represent higly stigmatized conditions where re-identification would cause significant harm to the affected individuals. Therefore, the only acceptable re-identification probability was zero. The resulting file had 2,375,331 records. In this section we provide the parameters for the PUMF.

#### Quasi-identifiers

The quasi-identifiers that were included in our analysis are summarized in Table 2. The different levels of precision, or the generalization hierarchy, for each variable are also included in the table. Diagnosis and intervention code details are provided in Additional file 1. The generalization hierarchy for diagnosis codes and intervention codes is based on a standard hierarchical coding scheme.

**Table 2:** The Quasi-identifiers

Quasi-identifiers	Coding	# Categories
PROV_XXX	Province/region. Quebec data is not included in the DAD.	
PROV_ALL	The territories are grouped into one category + 9 provinces	10
PROV_REGION	The country is divided into three regions (West, Central, and East), where Central consists of Ontario.	4
TOTAL_LOS_XXX	Total Length of stay	
TOTAL_LOS_DAYS	Days up to 1 week, then in weeks up to 6 months, and top coded at 6 months +	31
TOTAL_LOS_WEEKS	Weeks up to 6 months everything longer than that is top-coded into a single category	25

AGE_GROUP	Five year intervals and top coded at 90 years	20

GENDER_CODE	unchanged	5^1^

MRDx DIAG3 DIAG_BLOCK DIAG_CHAPTER	Different levels of coding detail of the most responsible diagnosis code.	8967 1435 195 23

CMG_CODE	These identify Case Mix Groups (CMGs), which are groups of patients with similar clinical and cost characteristics. They are based on most responsible diagnosis (MRDx) and other diagnosis and intervention information.	545

CCI_CODE SHORT_CCI	Different levels of coding detail of the principle intervention. Approximately 46% of the records had no interventions.	8780 569

This table displays the quasi-identifiers that were being analyzed and the number of response categories. There are two versions of the province and total length of stay fields as they represent different levels of detail.

^1 ^Five gender codes are used in the DAD: F-female, M-male, O-other (trans-sexual or hermaphrodite, U-undifferentiated(stillbirths only), and Z-missing.

The generalization hierarchies defined in Table 2 were created in consultation with experts at the data custodian who regularly perform analysis on discharge abstract data. These experts considered the utility of the data from the perspective of multiple data users. For example, a PUMF that is useful for educational and training purposes at a college or university course may not be useful for a complex health services research study. Examples of the types of end-uses considered were: education and training of students and fellows, preparation of analysis plans before making data access requests for the full DAD, and evaluation data sets for computer scientists and statisticians when developing new algorithms and modeling techniques.

The generalizations in Table 2 represent meaningful generalizations of the quasi-identifiers that would still retain some utility for analysis. Any further generalizations were deemed not to be acceptable from the perspective of the data users. Intervals for the continuous variables were chosen to be analytically meaningful.

The generalization hierarchies also represent policy decisions made by the organization around the granularity of information that they are willing to disclose. For example, the disclosure of postal codes or patient location information more specific than province was not acceptable to CIHI.

#### Focus of PUMF

We evaluated two different types of PUMFs: (a) a *geographic PUMF *which contains the patients' province but reduced clinical quasi-identifiers (no length of hospital stay information), and (b) a non-geographical *clinical PUMF *where the lack of geographic detail would allow for including length of stay. The two data sets that were created are summarized in Table 3. Each PUMF represented a different non-overlapping 10% random sample from the population DAD data set and so would included approximately 240,000 records.

**Table 3:** The two PUMF data sets that would be created

ID	PROV_XXX	TOTAL_LOS_XXX	AGE_GROUP	GENDER_CODE	MRDx	CCI_CODE
1: Geographic PUMF	X		X	X	X	X

2: Clinical PUMF		X	X	X	X	X

#### Characteristics of Adversaries

It is important to understand the characteristics of the potential adversaries who can attempt to re-identify individuals in the PUMF. This will help us determine the plausibility of different attacks and direct us in choosing appropriate metrics for measuring the probability of re-identification. When making data available as a PUMF one needs to make the worse case assumptions about the adversary because it is difficult to predict who would make a re-identification attempt. Consequently, we assumed that the adversary has unlimited financial resources and time, and is by definition not bound by any data sharing agreements that would constrain his/her behaviour.

#### Measuring the Probability of Re-identification

The primary metric we used to evaluate how likely it is for an adversary to re-identify one or more individuals in the PUMF is the probability of correct re-identification. This is measured by 1/*k *within the context of the k-anonymity criterion. We do not consider the probability of incorrect re-identification in our evaluations. This is common practice, thus far, in the disclosure control community.

If the adversary matches, say, 1,000 records to a population registry and is able to correctly re-identify only 50 records, a data custodian may consider that to be acceptable. But there will be 950 records that were incorrectly re-identified. Would this be acceptable?

If the adversary will be sending email spam to these 1,000 re-identified patients then one can argue that there is no substantive injury to the 950 patients. If the adversary was going to try to fraudulently use the health insurance policies of the 1,000 patients or open financial accounts in their names, there will still be no injury to the 950 patients (because the re-identified identities will not match the actual identity information during the verification process, and therefore these attempts will very likely fail). Under such conditions we would not consider the incorrect re-identifications as a problem.

On the other hand, if a reporter was going to expose a person in the media based on incorrect information then this could cause reputational harm to the patient. Or if employment, insurance, or financial decisions were going to be made based on the incorrectly re-identified data then there could be social, psychological, or economic harm to the individuals.

If we make the probability of incorrect re-identification sufficiently high for the adversary, then we argue that this would act as a deterrent to launching a re-identification attack. This argument is based on two assumptions: (a) an incorrect re-identification is detrimental to the adversary's objectives, and (b) information about the probability of re-identification of individuals in the PUMF is made public so that it is known to the adversary to influence the adversary's behaviour.

Consequently in this paper we will only talk about the probability of re-identification to mean the probability of correct re-identification.

#### Probability of Re-identification Threshold

We need to define the probability of re-identification threshold. This represents the maximum probability that the custodian is willing to accept when disclosing the PUMF [112]. Some broad guidelines have been developed specifically for the creation of PUMFs within a Canadian context [113], however these did not recommend any specific thresholds.

There is considerable precedent for a probability threshold of 0.2, which is often expressed in terms of a "cell size of five" rule (or k = 5) [22,114-122]. However, this threshold is more commonly used when data is disclosed to a trusted entity, such as a researcher, rather than for disclosing data to the public. It is also used when the data recipient will sign a data use/sharing agreement with the custodian. In some cases a threshold as high as 0.33 is used (a cell size of three) [123-126], although in practice this would be under quite restrictive conditions.

In the US, the Safe Harbor standard in the HIPAA Privacy Rule is sometimes used as a basis for the creation of PUMFs since such data is no longer considered protected health information. It has been estimated that Safe Harbor implies that 0.04% of the population is unique (has k = 1) [127,128]. Another empirical re-identification attack study evaluated the proportion of Safe Harbor records that can be re-identified and found that only 0.01% can be correctly re-identified with certainty [44]. However, it can be argued that using a k value that is so low would not be acceptable for a PUMF, especially with the adversary profile that we described earlier.

In the current analysis we considered two probability thresholds, 0.04 (i.e., k = 25) and 0.05 (i.e., k = 20). The data custodian deemed these thresholds to be acceptable to the organization.

### Possible Re-identification Attacks

In order to reduce the re-identification probability of the data set properly we had to understand the potential attacks that an adversary may attempt. Assuming that the adversary does not verify matches, there are three possible attacks which will be described below.

**Figure 1:**
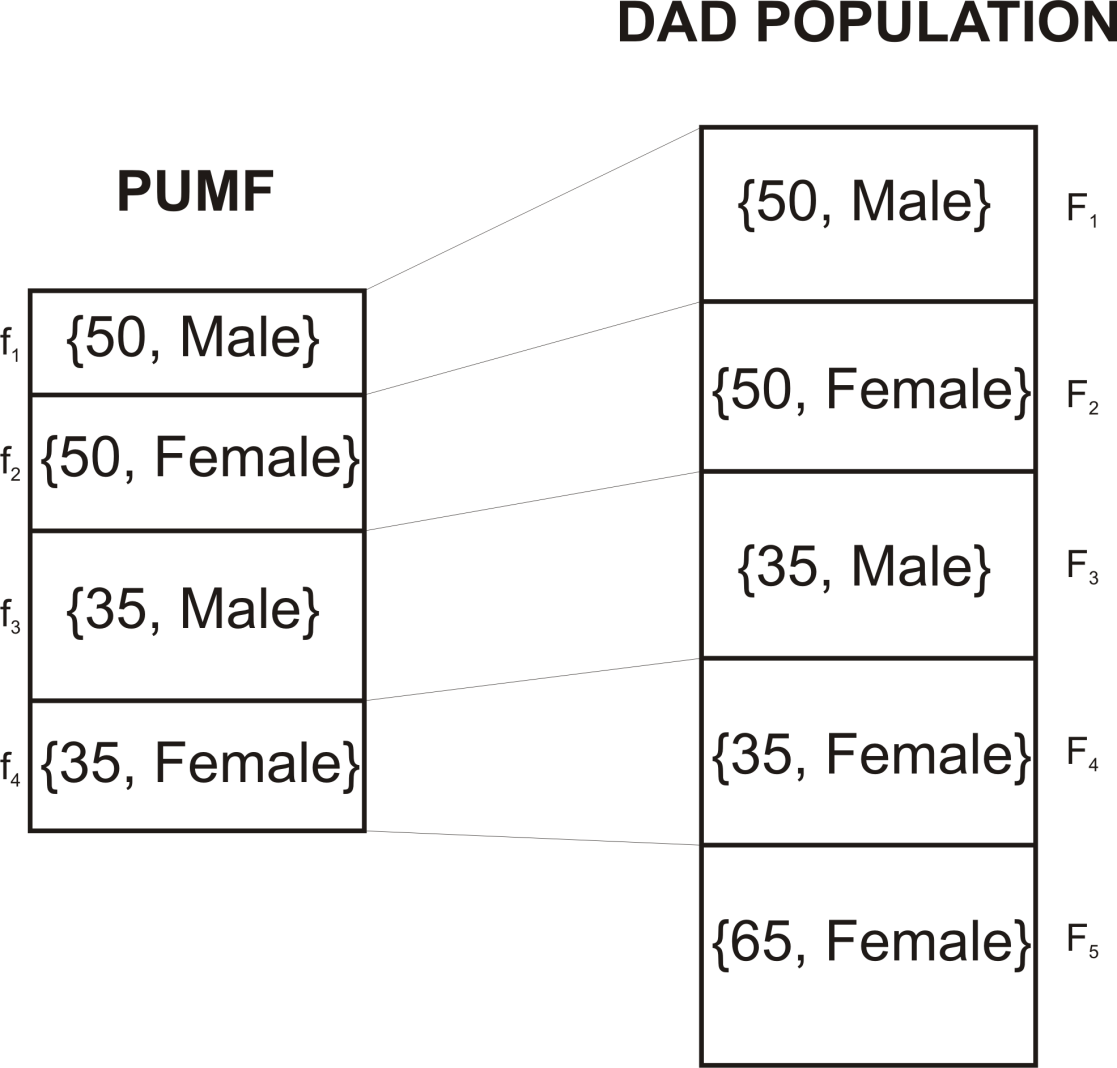
**Notation for equivalence classes**. This example shows a PUMF with 4 equivalence classes sampled from a population data set with 5 equivalence classes. The PUMF set has four elements: [50, Male], [50, Female], [35, Male], [35, Female]. The *f_j _*and *F_j _*denote the equivalence class sizes for the PUMF and DAD population data sets respectively. Note that in this example *f*_5 _= 0.

The notation we use is illustrated in Figure 1. This example shows a PUMF with 4 equivalence classes sampled from the DAD population data set with 5 equivalence classes. Let the set of DAD population equivalence classes be denoted by *K *and *j *∈ *K*. Let the set of equivalence classes in the PUMF be denoted by *J *where *J *⊆ *K*, and *f_j _*is the size of an equivalence class *j *in the PUMF and *F_j _*is the size of an equivalence class in the DAD population. Also note that f_j _≥ 0 and F_j _> 0. In the example of Figure 1, the *J *set has four elements: < 50, Male>, < 50, Female>, < 35, Male>, < 35, Female>.

An important assumption is that the adversary would not know which individuals are in the PUMF. This is reasonable since the PUMF would be a random sample from the population DAD. Therefore, even if an adversary knew that Alice was hospitalized in 2008, it would not be possible to know if she was selected into the PUMF. If the adversary knew who was in the PUMF then the probability of re-identifying a single individual would be 1/*f_j_*, which will typically be quite high for, say, a 10% sampling fraction (since *E(f_j_) *= 1 × *F_j_*). However, given that the adversary would not know who is in the PUMF, the actual probability of re-identification would be smaller than 1/*f_j_*.

#### Attack 1: Adversary Matches a Single Patient to a Registry

In this attack, the adversary has access to a public population registry containing identifying information and can use that to match against the PUMF. For an equivalence class that exists in both the PUMF and the population registry, the adversary then selects a record at random from either data set and matches it with a record in the other data set.

Previous research has suggested that it is relatively easy to construct population databases useful for re-identification about certain sub-populations in Canada because of the information available about them in public and semi-public registries [129,130]. As illustrated in Additional file 2, it is possible to create population registries for these specific sub-populations: professionals such as doctors and lawyers because their professional associations publish complete membership lists, homeowners because the Land Registry publicly maintains their names, and civil servants because the government publishes lists of government employees. These sub-populations are considered the at-risk members of the Canadian population from a re-identification perspective.

Re-identification under this attack could only occur if the population registry overlaps with the PUMF (i.e., there are individuals in both data sets). This is different from the commonly cited US examples where voter registration lists are available in many states [131]. In the US case a disclosed data set is often represented as a sample from the voter registration list and consequently the probability of re-identification would be measured differently.

**Figure 2:**
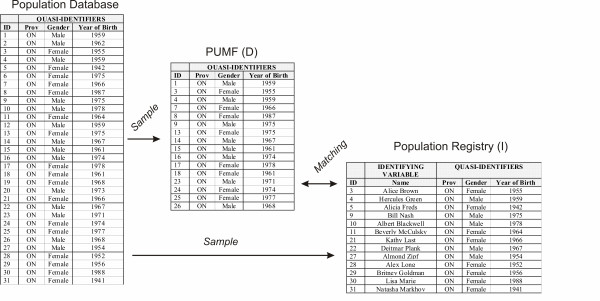
**Matching the PUMF against a population registry**. This figure shows the process that would be used in by an attacker. The ID field is only included here to make it easier to see which records are included in each data set. In reality there would be no consistent ID field available across the data sets.

The quasi-identifiers in the PUMF that also exist in publicly available population registries in Canada are age, gender, and province. Therefore, the matching exercise would be as shown in Figure 2.

Assuming there is an overlap between the individuals in the PUMF and the individuals in the population registry, then the probability of re-identification for any individual in equivalence class *j *in the PUMF is derived in Additional file 3 to be 1/*C_j _*where *C_j _*is the size of the equivalence class in the provincial population (measured through the census, for example). We consider provincial populations because that is the smallest geographic granularity in the PUMF. In Additional file 3 we also demonstrate the accuracy of this derivation using a series of matching experiment simulations (for example, see [132,133]).

The measure for the overall PUMF is the proportion of records that have a probability of re-identification higher than a threshold:

(1) $$\frac{1}{N_{p}}\underset{j\in J}{\sum }C_{j}\times I\left(\frac{1}{C_{j}}>\tau \right)$$

where *N_p _*is the population of the province, *I*( ) is the indicator function returning one if the logical parameter is true and zero otherwise, and τ is some maximum acceptable probability threshold (as noted earlier, this would be 0.04 or 0.05). The proportion in equation (1) was computed for every province and territory separately.

To evaluate equation (1), we used the data from the 2001 census PUMF from Statistics Canada. Four population subsets in the census matched our three at-risk groups: homeowners, federal government employees, healthcare professionals, and business professionals. For each subset within each province we computed the number of (weighted) individuals within each age and sex equivalence class that is smaller than the threshold. This amounted to 11 provinces and territories (all territories were grouped into a single category), four data subsets, and two thresholds, which was equal to 88 different analyses. In none of these analyses was the proportion of a province's population with a re-identification probability higher than 0.04 greater than 0.001.

Our results indicate that the proportion of the provincial populations that have a probability of re-identification on the quasi-identifiers that can be used for matching with public registries is small and therefore the chances of a successful re-identification using this attack is negligible.

#### Attack 2: Adversary Matches All Patients Against a Registry

In this attack the adversary also obtains a population registry as in Figure 2, however he then matches *all *individuals in the PUMF against the individuals in the registry. This is different from the attack above which focuses on the probability of re-identification for a single randomly selected individual.

Also, note that to launch this attack an adversary needs to obtain or create a sufficiently large population registry to have an overlap of individuals with the DAD PUMF. This can be quite expensive and therefore there is a potential economic deterrent for this attack. To construct a large population registry that is detailed enough can be costly in practice. For instance, to create a complete population registry with records containing names, home addresses (including postal codes), gender and date of birth for the 23,506 registered practicing physicians in Ontario (a professional group) at the time of the study would cost at least C$188,048. Similarly for the 18,728 registered lawyers, the cost is estimated to be at least C$149,824 [129]. Although under our assumptions, an unconstrained adversary could still spend that amount on a re-identification attempt.

With reference to Figure 2, let the adversary have the PUMF (data set *D*) and a registry (data set *I*). Let *D *and *I *be two sets representing simple random samples of individuals from a population (say, represented by the census). Note that *D *and *I *must have common records but that *I *is not necessarily a subset of *D *or vice versa, in other words, there is a possible overlap between the two for any matching to be successful: *D *∩ *I *≠ ∅. Assume that the set of population equivalence classes is denoted by *Q*, and *j *∈ *Q*.

In Additional file 3 we show that the proportion of records in the PUMF that can be correctly linked using an exact matching method with an overlapping registry is at most given by:

(2) $$\underset{j\in Q}{\sum }\frac{f_{j}}{C_{j}}$$

In Additional file 3 we also demonstrate the accuracy of the derivation using a series of matching experiment simulations (for example, see [132,133]). If we let *α *represent the sampling fraction of *D *from the population (census), then equation (2) can be re-written as:

(3) $$\underset{j\in Q}{\sum }\frac{\alpha C_{j}}{C_{j}}=\left|Q\right|\alpha$$

As noted above, population registries in Canada only include age, gender, and location information. Therefore, the quasi-identifiers of relevance for this kind of matching are: GENDER_CODE, AGE_GROUP, and PROV_ALL.

In our data set we can compute the maximum value of $\left|Q\right|$ as: 2 × 20 × 10 = 400. We only consider two values for GENDER_CODE because population registers do not account for the other three gender categories in the DAD. We can also compute *α*. According to Statistics Canada, the population of Canada in 2008 (excluding Quebec) was 25,573,800. Therefore a 10% sample from the PUMF would mean that *α *= 0.009, and approximately 4 individuals in the PUMF would be expected to be correctly re-identified according to equation (3). Furthermore, the adversary would not know which 4 individuals out of the .24 million in the file were correctly re-identified.

Given the low number of individuals that can be correctly re-identified and the adversary not knowing which individuals were correctly re-identified, we consider the chances that an adversary would try this attack, and succeed if attempted, to be negligible.

#### Attack 3: Adversary Targets a Specific Patient

Under this attack the adversary has background information about a specific individual, say a 35 year old male who is the adversary's neighbour. The adversary does not know if that target individual is in the PUMF. If the adversary does find an equivalence class *j *that matches the targeted individual, the adversary would then choose one of the *f_j _*records in that equivalence class at random as a match. The probability that the match is a correct one is 1/_Fj _[134]. Therefore, to maintain the probability threshold at or below 0.04, we must ensure that *F_j _*≥ 25 for all *j*through generalization and suppression, and to maintain a probability of 0.05 we must ensure that *F_j _*≥ 20 for all *j*.

This attack is plausible and represents the one that we needed to focus on. Therefore, the remainder of the paper describes how we created versions of a DAD PUMF that protect against this attack through generalization and suppression. Since we have the population DAD, generalization and suppression were performed on the full population DAD and the PUMF samples drawn from that.

### Strategies for Improving Data Utility

#### Level of Detail of Quasi-identifiers

Under attack number 3, our archetype adversary who would attempt to re-identify records in the PUMF is a neighbour. For any patient Alice, a neighbour would know the basic demographics of Alice (GENDER_CODE, AGE_GROUP, and PROV_ALL). It is also very plausible that Alice would tell her neighbour why she went to the hospital and what the primary interventions were. Therefore, it is plausible that this kind of information can be obtained by such an adversary.

Sometimes the adversary will know the full detail for a quasi-identifier that can be hierarchically generalized. Length of stay is such an example: a patient's neighbour can notice how many days the patient was in the hospital.

However, it is unlikely that an adversary would be able to determine the full ICD-10 (International Statistical Classification of Diseases and Related Health Problems, 10^th ^Revision) [135] code for the most responsible diagnosis as these tend to be very specific and include some clinical details that Alice herself may not know with such specificity. Similarly, Alice will likely only provide a high level description of the intervention. The level of detail at which an adversary would know the quasi-identifiers, such as the diagnosis, can help us set limits on the amount of generalization that needs to be performed.

**Figure 3:**
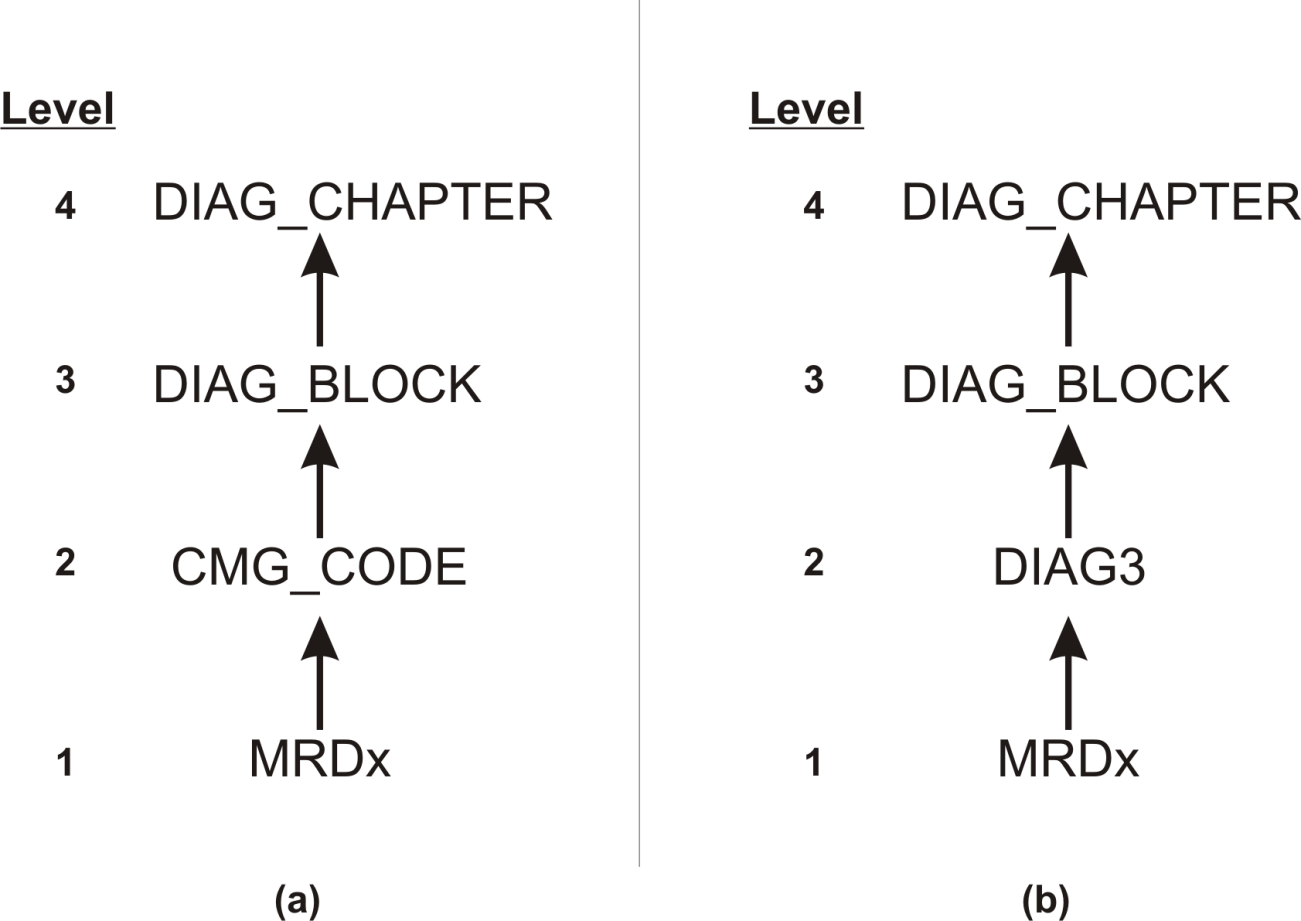
**An example of a domain generalization hierarchy for the diagnosis information**. In this example we can see that at the lowest level (level 1) this variable is disclosed with the most detail: the actual ICD-10 most responsible diagnosis. This can be generalized into the CMG_CODE (panel a), which is not a simple generalization from MRDx, but also considers other information about the acute admission (level 2). The MRDx value can also be generalized to a three character code DIAG3 (panel b), which is a direct generalization in the ICD-10 hierarchy. The diagnosis can be further generalized to the ICD-10 block (level 3), and then the chapter (level 4).

Let us consider the diagnosis variable and the diagnosis generalization hierarchy shown in Figure 3. This hierarchy has four levels. In this particular case the domain generalization hierarchy is not totally ordered [82] because multiple MRDx codes can generalize to a single CMG_CODE, and a CMG_CODE can be specialized into multiple possible MRDx codes.

If we assume that the adversary is not likely to know the full ICD-10 code for MRDx of the patient, then it need not be considered a quasi-identifier (at least for this adversary) except insofar as it could be used to derive a more general version of diagnosis that is known to the adversary and therefore useful as a quasi-identifier. For instance, if the adversary is likely to know the CMG_CODE for a patient then we need to apply de-identification at that level in the generalization hierarchy, and to all generalizations of CMG_CODE as well. In addition, it would be necessary to ensure that suppressed CMG_CODE values could not be derived from the MRDx codes; in general if a CMG_CODE value was suppressed, the MRDx values that generalize to the suppressed CMG_CODE value would have to also be suppressed. Similarly, it would be necessary to ensure that suppressed DIAG_BLOCK values could not be derived from the MRDx codes and therefore the MRDx values that generalize to a suppressed DIAG_BLOCK value would have to be suppressed as well. We will refer to this as a propagation of suppression from one quasi-identifier to another.

Using the general rule set in Table 4 and our example, we would suppress quasi-identifiers assuming level 2 knowledge (and its generalization to levels 3 and 4), and propagate these suppressions to all values at level 1.

**Table 4:** Rules to use for constraining the search for the de-identification solution

Relationship between maximum acceptable generalization (M) and adversary's background knowledge (Q)	Generalization level to use for suppression (S)	Generalization level to release data at
*M *≥ *Q*(analysts could accept a generalization equal to or less detailed than the adversary's knowledge)	*S *= *M *(suppress at level M)	Only include data generalized to level *S *= *M*

*M *<*Q *(analysts need a version that is more detailed than the adversary's knowledge)	*S *= *Q*	The PUMF can have data at level *M *in the generalization hierarchy, except when it generalizes to a suppressed value at level *S *= *Q*.

The generalizations on the right-hand column can be applied to each quasi-identifier separately. The symbol *M *denotes the generalization hierarchy level representing the highest generalization level acceptable for analysis. The symbol *Q *denotes the generalization hierarchy level representing the adversary's background knowledge, and *S *denotes the level at which the suppression should be performed. (In our example, M = 1, and *Q *= 2. Therefore, using this example, the *M *<*Q *condition is met and we should: suppress at level *Q*; and include partially suppressed data at level *M*).

With this approach it may be possible to disclose more detailed information and minimize suppression. However, the assumptions about the level of detail of the adversary's knowledge, and so which levels in the hierarchy need to be considered as quasi-identifiers, must be defensible.

The general principle described here can be applied in the case of simple domain generalization hierarchies. For example, the generalization hierarchy in panel (b) of Figure 3 shows a diagnosis code hierarchy whereby each MRDx value generalizes to a single DIAG3 value, where DIAG3 consists of the first three characters of the diagnosis code. Previous work has shown that this more common generalization hierarchy for diagnosis codes (as depicted in panel b) can result in excessive suppression [136]. Therefore in the current study we only consider the generalization hierarchy in panel (a) of Figure 3.

#### Disclosing All Levels of the Hierarchy

To maximize utility to the user of the data, we can include all levels of the quasi-identifier hierarchy in the PUMF. In this manner the data user can select the level of precision most suitable for their analysis and there is no need to find a single optimal generalization for all types of users.

We need to evaluate the probability of re-identification and apply suppression for each level in the hierarchy separately. Consider the example of the most responsible diagnosis code. The adversary would have background knowledge at a specific level, say MRDx. Then the adversary can attempt re-identification using the combination in 1a of Table 5 for the geographic PUMF. If the adversary knew the most responsible diagnosis at a different level, say CMG_CODE then s/he would attempt to re-identify with the combination in 1b. The probability of re-identification has to be measured four times. In the case of 1a, only the demographic variables and MRDx would be included in the measurement. Appropriate suppression would then be performed on the demographics and MRDx to ensure that the probability of re-identification on these four quasi-identifiers is at or below the threshold. If the adversary knew diagnosis at the CMG_CODE level, then CMG_CODE is included with the demographic quasi-identifiers, as in analysis 1b. Then in analysis 1c, the demographics would be combined with the DIAG_BLOCK, and suppression applied for the equivalence classes that are too small. Once all of the four cycles of suppression have been completed, all seven variables can be included in the PUMF.

**Table 5:** The four sets of evaluations that would be performed at each level of the most responsible diagnosis code

Combination ID	PROV_XXX	AGE_GROUP	GENDER_CODE	MRDx	CMG_CODE	DIAG_ BLOCK	DIAG_ CHAPTER
**1a**	X	X	X	X			

**1b**	X	X	X		X		

**1c**	X	X	X			X	

**1d**	X	X	X				X

For each value of diagnosis code there is a combination of quasi-identifiers that the adversary can use.

Using the scheme above, any combination of demographics and a diagnosis code will have a probability of re-identification below the threshold. Therefore, we protect against all possible levels of background knowledge that the adversary may have. However, we make available the very detailed diagnosis code information as well.

To implement this scheme effectively we needed to develop an appropriate suppression algorithm. Consider the example in Table 6, which assumes that we must ensure that each equivalence class must be of size 2 (i.e., 2-anonymity). Here all equivalence classes on the combination < PROV_ALL, AGE_GROUP, GENDER_CODE, MRDx> are of size two and all equivalence classes on the combination < PROV_ALL, AGE_GROUP, GENDER_CODE, CMG_CODE> are of size two. If we applied a suppression algorithm on each of the two combinations of quasi-identifiers there would be no suppression. This is the best result because an adversary would try to re-identify using one of the two combinations. For example, if the adversary had MRDx, then s/he would attack the data set with the combination < PROV_ALL, AGE_GROUP, GENDER_CODE, MRDx>. Even if the adversary had MRDx and CMG_CODE s/he would still attack the data set with the < PROV_ALL, AGE_GROUP, GENDER_CODE, MRDx> combination because it will be the most specific and have the smallest equivalence classes.

**Table 6:** Example of a data set with MRDx and CMG_CODE

PROV_ALL	AGE_GROUP	GENDER_CODE	MRDx	CMG_CODE
ON	[50-59]	M	B022	013

ON	[50-59]	M	B022	033

ON	[50-59]	M	C793	013

ON	[50-59]	M	C793	033

A heuristic algorithm for suppressing combinations is described below. This algorithm iterates through all of the combinations and applies a heuristic suppression method on each. In our example it would iterate through < PROV_ALL, AGE_GROUP, GENDER_CODE, MRDx> and < PROV_ALL, AGE_GROUP, GENDER_CODE, CMG_CODE> in turn and apply suppressions.

The basic principle of the scheme accounting for multiple combinations of quasi-identifiers can be applied with multiple quasi-identifiers whose levels will be disclosed. Table 7 shows the results for when we have two hierarchical quasi-identifiers and hence there are eight possible combinations of background knowledge that an adversary would have. In such a case the suppression algorithm would iterate through the eight combinations.

**Table 7:** Example showing the eight combinations when we have two hierarchical quasi-identifiers where we disclose all of their levels

Combination ID	PROV_XXX	AGE_GROUP	GENDER_CODE	MRDx	CMG_CODE	DIAG_BLOCK	DIAG_CHAPTER	CCI_CODE	SHORT_CCI
**2a**	X	X	X	X				X	

**2b**	X	X	X		X			X	

**2c**	X	X	X			X		X	

**2d**	X	X	X				X	X	

**2e**	X	X	X	X					X

**2f**	X	X	X		X				X

**2g**	X	X	X			X			X

**2h**	X	X	X				X		X

For hierarchical quasi-identifiers where the hierarchy is totally ordered, further combinations can be skipped. For example in Table 5, the suppressions in DIAG_BLOCK performed on combination 1c can just be propagated to DIAG_CHAPTER without having to evaluate combination 1d.

Furthermore, this approach can be combined with the earlier one (having all levels of detail for a quasi-identifier) to minimize the amount of suppression. For example, if we assume that the adversary would only have background knowledge at the CMG_CODE level, then we would skip the suppressions in 1a, and apply the same suppressions on CMG_CODE from cycle 1b to the MRDx variable. This would reduce the amount of suppression in MRDx significantly.

For a more complete example, consider Table 8 which shows that only two combinations would need to be considered if we assume that the adversary has knowledge at the CMG_CODE level for diagnosis code and SHORT_CCI for the intervention variable. Then the suppressions on CMG_CODE would have to be propagated to MRDx, the suppressions on SHORT_CCI propagated to CCI_CODE, and the suppressions on DIAG_BLOCK propagated to DIAG_CHAPTER.

**Table 8:** Example showing the two combinations when we have two hierarchical quasi-identifiers where we disclose all of their levels but assume that the adversary would only have background knowledge at the CMG_CODE and SHORT_CCI levels, and where one of the hierarchies is totally ordered

Combination ID	PROV_XXX	AGE_GROUP	GENDER_CODE	MRDx	CMG_CODE	DIAG_BLOCK	DIAG_CHAPTER	CCI_CODE	SHORT_CCI
**2f**	X	X	X		X				X

**2g**	X	X	X			X			X

### Suppression Algorithm for Combinations

#### Current Approaches

We needed an algorithm that was suitable for suppressing combinations. There are three general approaches to suppression: casewise deletion, quasi-identifier removal, and local cell suppression [137].

Casewise deletion removes the whole record from the data set. This results in the most distortion to the data because the sensitive variables are also removed even though those do not contribute to an increase in the probability of identity disclosure. Casewise deletion would not apply to combinations of quasi-identifiers as it would remove all of the record.

Quasi-identifier removal only removes the values on the quasi-identifiers in the data set. This has the advantage that all of the sensitive information is retained. However, it would not apply to combinations because all of the quasi-identifiers would be removed.

Local cell suppression is an improvement over quasi-identifier removal in that fewer values are suppressed. Local cell suppression applies an optimization algorithm to find the least number of values ("cells") on the quasi-identifiers to suppress. All of the sensitive variables are retained and in practice considerably fewer of the quasi-identifier values are suppressed compared to casewise and quasi-identifier deletion. We therefore consider algorithms for local cell suppression that will ensure k-anonymity.

One existing suppression algorithm has a 3*k*(1 + log2*k*) approximation to k-anonymity with a *O*(*n*^2*k*^) running time [138], where *n *is the number of records in the data set. Another had a 6*k*(1 + log *m*) approximation and *O*(*mn*^2 ^+ *n*^3^) running time [138], where *m *is the number of variables. An improved algorithm with a 3(*k*-3) approximation has been proposed [139] with polynomial running time. Because of its good approximation, the latter algorithm has been used in the past on health data sets [87,140].

However, all of the above suppression algorithms require the computation of a pairwise distance among all pairs of records in a data set. This means *n*(*n*-1)/2 distances need to be computed because they require the construction of a complete graph with *n *vertices and the edges are weighted by a distance metric (the number of cells that differ between the two nodes). Such algorithms can realistically only be used on relatively small data sets.

Furthermore, these algorithms do not account for combinations and can only be used on all of the quasi-identifiers in the data set at once. For example, if we apply suppression on all of the five quasi-identifiers in Table 6 we would have to suppress either all of the values in MRDx or all of the values in CMG_CODE. By considering the combinations seperately less suppression would be necessary. Therefore, we need to use a suppression algorithm which would determine the suppressions required for each combination of quasi-identifiers separately. Below we describe such an algorithm.

#### Combinations Suppression Algorithm

In this section we describe a heuristic algorithm for local cell suppression when we have overlapping combinations of quasi-identifiers. We assume that the data set has *n *records and *i*∈{1,...,*n*}. Let **B **be the set of quasi-identifier combinations, and b ∈ **B**. Each combination has a fixed number of quasi-identifiers denoted by the set **Z***_b_*, and let *z*_b _∈ **Z***_b _*(which means that *z_b _*is a quasi-identifier in that particular combination). A single cell in the data set can be indexed by *V*[*i, z_b_*] which indicates the record, the specific combination and quasi-identifiers in the combination.

The possible values for each quasi-identifier are represented by the set ***L***[*z_b_*]. For example, if the quasi-identifier is AGE_GROUP then it would have 20 possible 5 age intervals. Let l[*z_b_*]∈ ***L*[***z_b_***] **be one of the quasi-identifier values. The support of *l*[*z_b_*] is the number of records that have that particular value: sup(*l*[*z_b_*]).

Individual quasi-identifiers may be assigned a weight. Weights have values between zero and one. A quasi-identifier with a higher weight would be affected less during suppression because it would be considered a more important variable. A weight is denoted by *w*[*z_b_*]. We then define the weighted support function as: wsup(*l*[*z_b_*]) = sup(*l*[*z_b_*]) × *w*[*z_b_*].

Let *E*(*l*[*z_b_*]) be a function which returns a set of all equivalence classes that have a value of *l*[*z_b_*]. If *x *∈ *E*(*l*[*z*]) then $\left|x\right|$ is the size of the equivalence class.

The data custodian has set a risk threshold, *τ*, and *k *=⌈1/*τ*⌉ represents the minimum equivalence class size needed to ensure that the risk is equal to or greater than *τ*. Let *M_b _*be the number of equivalence classes smaller than *k *for a particular combination. These equivalence classes are called *moles*.

The algorithm has two general phases. In the first phase it removes all values in the quasi-identifiers that have infrequent support (i.e., support less than *k*). The reasoning is that these values by definition will always be problematic and therefore have to be removed. In the second phase the algorithm iterates through each quasi-identifier to remove the values with minimal support.

**Figure 4:**
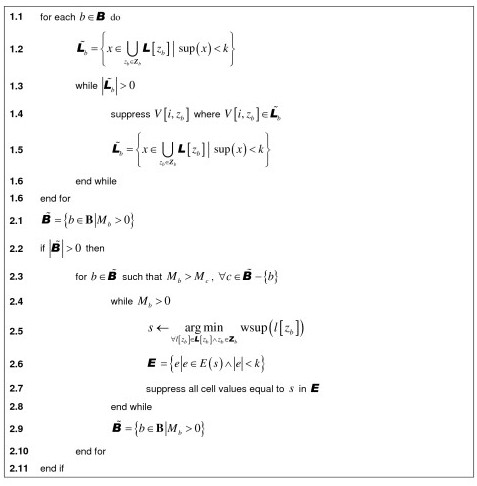
**Pseudocode for the suppression algorithm**.

The algorithm is shown in Figure 4. It iterates through the combinations (step 1.1). For each combination, it determines which quasi-identifiers have values with infrequent support (step 1.2). The values that have infrequent support are then suppressed (step 1.4).

We then identify the combinations with moles (step 2.1). The quasi-identifier combinations with moles will be ordered with combinations having the largest number of moles first. The algorithm then iterates through these combinations with moles in order (step 2.3). Within each combination we find the value on any of the quasi-identifiers that has the smallest weighted support (step 2.5). This value is then suppressed from all equivalence classes that are smaller than *k *(steps 2.6 and 2.7). This is repeated until there are no more moles in the combination, and then we proceed to the next combination.

#### Suppression Algorithm Walkthrough

**Figure 5:**
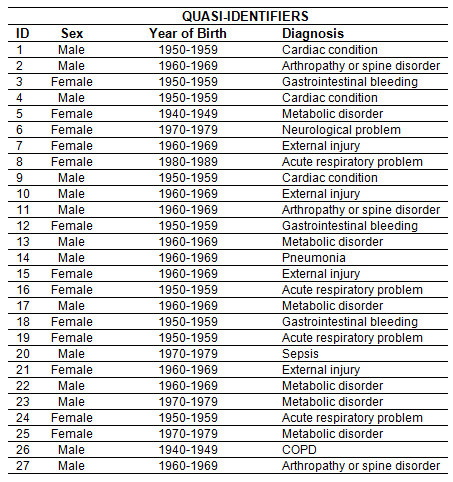
**Example data set for suppression algorithm walkthrough**.

We will illustrate how the suppression algorithm works through a small example. To simplify the presentation we will consider only one combination as exactly the same steps would be repeated for each combination in the data set. The example data set we will use is shown in Figure 5. This has threee quasi-identifiers: sex, year of birth grouped into ten year intervals, and the primary diagnosis. We will assume that we are aiming for *k *= 3 and the weight is one.

**Figure 6:**
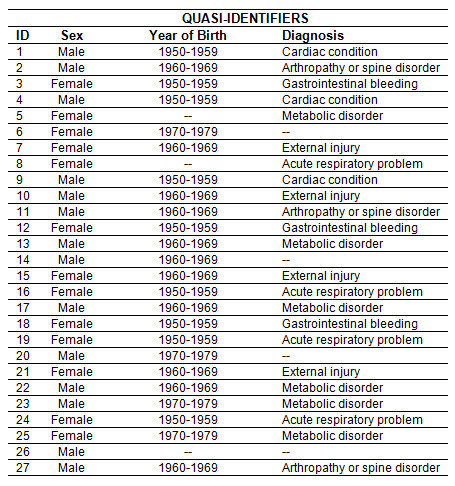
**Suppressions after the first phase of the suppression algorithm**.

In the first phase of the algorithm we identify the values for the quasi-identifiers that have a support less than *k*. For the sex quasi-identifier there are no sauch values. For the year of birth variable two values have a support less than three: 1940-1949 has a support of 2 (ID 5 and 26) and 1980-1989 has a support of 1 (ID 8). Therefore these three values would be suppressed. The diagnosis quasi-identifier as four values that have a support of 1: COPD, sepsis, neurological problem, and pneumonia. The final suppression after the first phase of the algorithm is shown in Figure 6.

**Figure 7:**
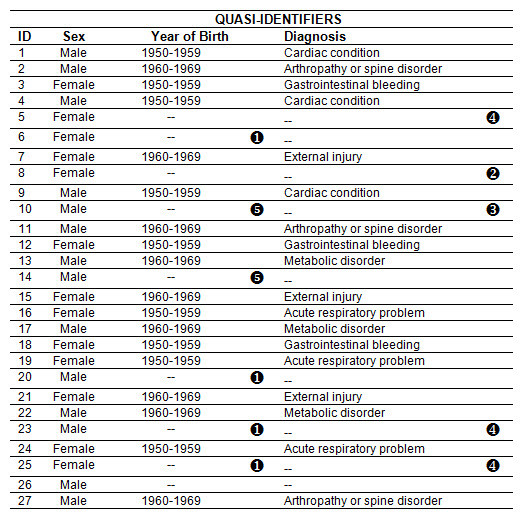
**Suppressions after the second phase of the suppression algorithm**.

In the second phase of the algorithm five passes of suppression are necessary, and these are labeled accordingly in Figure 7. We start with the value having the smallest support. A diagnosis of "cardiac condition" has a support of 3. We then identify all equivalence classes smaller than 3 that have this diagnosis. In this case there are none. Similarly, there are no small equivalence classes with a diagnosis of "arthropathy or spine disorder" or "gastrointestinal bleeding".

The next smallest support is 4. A birth year in 1970-1979 has a support of 4. All equivalence classes with this year of birth are smaller than 3, and therefore this value is suppressed from all the small equivalence classes within which it appears. These are labeled by a (1) in Figure 7. Next the diagnosis of "acute respiratory problem" has a support of 4, and it appears in an equivalence class of size one (ID 8). That diagnosis value is therefore suppressed within that equivalence class and is labeled with a (2) in Figure 7. The other equivalence class where this diagnosis appears has a size of 3 (ID 16, 19, and 24), and therefore will not undergo any suppression. A similar process is followed for the other value with a support of 4: "external injury".

We next consider a support of 6, which is a diagnosis of "metabolic disorder". There are two equivalence classes of size 1 with this diagnosis, and therefore the diagnosis is suppressed in these (ID 23 and 25).

The next support value is 9 for a year of birth in 1950-1959. All equivalence classes with this value are of size 3, and therefore will not undergo any suppression.

The next support is 11 for the of birth of 1960-1969. Here we have an equivalence class of size 2 (ID 10 and 14), in which case the year of birth will be suppressed.

At this point there are no longer any values on the quasi-identifiers that are smaller than 3, and the algorithm stops for this combination.

#### Computational Complexity of Suppression Algorithm

Our complexity analysis makes worse case assumptions, and we assume that always *k *> 1.

For phase 1 of the algorithm we assume that all values on all quasi-identifiers have low support. Computation of this support can be done once for all combinations. The computation of support requires a scan of all of the records. Therefore, the initial computation of support requires $\left|\underset{b\in B}{⋃}Z_{b}\right|\times n$ operations. During that step we would also keep index information about the records with each support value. Under a worse case scenario $\left|{\tilde{L}}_{b}\right|=n\times \left|Z_{b}\right|$ for all *b *∈ ***B***. This means that every value in the data set is unique. Therefore it would be necessary to iterate that many times to perform suppressions. The supressions would use the stored index values to avoid performing additional scans, but would still need to perform *n *suppressions. The total number of computations for phase 1 would then be $3\times \left|\underset{b\in B}{⋃}Z_{b}\right|\times n$. This will be dominated by the *n *term given that $n\gg \left|\underset{b\in B}{⋃}Z_{b}\right|$ in practice.

For the second phase we would need to compute the support for each value, but for this we would use the support values computed and updated during phase 1 of the algorithm. However, we will still need to compute the equivalence class sizes for each combination, which requires $\left|Z_{b}\right|\times n$ operations. In the worse case scenario we would need to examine every value because it would have low support, and suppress it in every equivalence class because they would all be small. This would give a total number of iterations equal to $\underset{z_{b}\in Z_{b}}{\sum }\left|L\left[z_{b}\right]\right|$. If each quasi-identifier has *n *possible values, then we would need $n\times \left|Z_{b}\right|$ iterations. At each iteration we need to update the affected equivalence class counts requiring at most a total of *n *computations. Also, at most we would perform $\left|Z_{b}\right|\times n$ suppressions in total. Therefore the total amount of computation in phase 2 would be $\underset{b\in B}{\sum }\left(3\times \left|Z_{b}\right|\times n+n\right)$ assuming all combinations had moles.

In general, we would expect the amount of computation to increase at most linearly with the data set size.

### Evaluation

Our empirical evaluation of the generated versions of the PUMF and the methods used generate them consisted of three components.

#### Measuring Information Loss

We assessed information loss using two parameters. The first was the extent of suppression. As noted earlier, suppression is an intuitive metric that data analysts can easily comprehend while assessing data quality. Suppression can be measured as: (a) the percentage of records that have some suppression in them (on the quasi-identifiers) due to the de-identification, or (b) as the percentage of cells in the file that are suppressed.

The second information loss metric we used was non-uniform entropy. Entropy was chosen because it has many desirable properties compared to other proposed information loss metrics in the literature. For example, non-uniform entropy will always increase when a data set is generalized and before suppression is applied (monotonicity property) and will behave in expected ways with data having an unbalanced distribution (see the review in [87]). Because entropy has no easily interpretable unit, we used one of the data sets as a baseline and compared the other data sets to that as a percentage increase in entropy.

#### Distribution of Missingness

The distribution of missingness due to suppression was also evaluated. For the selected PUMF we evaluated whether diagnosis codes are more likely to be suppressed based on age, region, and length of stay. For example, if we take the province variable, equivalence classes in Ontario will tend to be larger than in Prince Edward Island (PEI). Therefore, the level of suppression for the diagnosis codes in Ontario would likely be less. A PUMF user may restrict his/her analysis to Ontario only if there is too much diagnosis code suppression in PEI. In such a case, knowledge of the distributions of suppressions would be useful.

#### Performance of Suppression Algorithm

We evaluated the performance of our combinations suppression algorithm, which will be termed the "combinations" algorithm.. To help interpret its performance, we compared it to an algorithm that does not take combinations into account, which will be termed the "complete" algorithm. The latter is the same algorithm as the former except that there is only one combination consisting of all of the quasi-identifiers.

The performance evaluation was conducted on a Windows machine with a dual core Intel processor running at 2.6 GHz and 2GB of RAM. Both suppression algorithms were implemented in the C# programming language.

Three sets of performance evaluations were performed. The first compared the overall algorithm performance in seconds on each of the four data sets. The second compared the overall time in seconds for each of the two phases of the algorithms. We varied the value of *k *from 3 to 40. This allows us to determine whether the performance of the algorithm is affected as the threshold for acceptable re-identification probability changes. Both algorithms are expected to perform more iterations as the value of *k *increases.

The third evaluation was to assess how the algorithm scales with the number of records in the data set. For each of our data sets we created random sub-samples with sampling fractions varying from 0.1 to 0.9 in increments of 0.1. For each sub-sample we computed the time to perform the suppression. The value of k was fixed at 5 for these evaluations.

## Results

### Information Loss

In our analysis we assumed that the adversary would only know the diagnosis code at the CMG_CODE level and not at the MRDx level. Similarly, we assumed that the adversary would only know the short version of the intervention code rather than the detailed intervention code.

The number of records with suppression on each of the quasi-identifiers for the geographic and clinical PUMF files (extent of missingness) are shown. We present the results at the alternate 0.04 and 0.05 probability threshold levels as shown in Table 9 and Table 10. We also include the 0.2 probability threshold as a baseline value for comparison (the 0.2 threshold is often used when there are factors that mitigate the risk, such as data use agreements with trusted agents).

**Table 9:** Main results for geographic file

	PROV_ALL						PROV_REGION					
	**0.2**		**0.05**		**0.04**		**0.2**		**0.05**		**0.04**	

	**Comb**.	**Complete**	**Comb**.	**Complete**	**Comb**.	**Complete**	**Comb**.	**Complete**	**Comb**.	**Complete**	**Comb**.	**Complete**

**GENDER_CODE**	0.002	0.002	0.005	0.005	0.005	0.005	0.002	0.003	0.003	0.009	0.002	0.012

**AGE_GROUP**	1.6	5.2	5.4	14.2	6.5	16.4	0.81	3.8	2.8	10.6	3.4	12.2

**PROV_XXX**	0.07	3.11	0.2	7.4	0.25	8.3	0.06	0.3	0.16	0.5	0.19	0.6

**MRDx**	12.6	14.4	26.8	27	29.4	29.4	8.6	10.7	19.4	21.4	21.6	23.5

**CMG_CODE**	12.6	14.4	26.8	27	29.4	29.4	8.6	10.7	19.4	21.4	21.6	23.5

**DIAG_BLOCK**	8.5	4.8	19.8	9.5	22.2	10.5	5.5	3.6	13.5	7.5	15.2	8.3

**DIAG_CHAPTER**	1.5	0.11	3.8	0.3	4.3	0.4	0.87	0.07	2.5	0.3	2.8	0.31

**CCI_CODE**	5.9	8.13	10.7	13.2	11.5	14.14	4.4	6.8	8.8	11.9	9.7	12.7

**SHORT_CCI**	5.9	8.13	10.7	13.2	11.5	14.14	4.4	6.8	8.8	11.9	9.7	12.7

**Total % Cells Suppressed**	**5.4**	**6.5**	**11.6**	**12.4**	**12.8**	**13.6**	**3.7**	**4.75**	**8.4**	**9.5**	**9.4**	**10.4**

**Entropy (%)**	**100**	**137**	**246**	**302**	**274**	**334**	**70**	**104**	**181**	**236**	**204**	**262**

Missingness (as a percentage of all records) for different probability threshold levels and levels of geographic detail for the quasi-identifiers in the first PUMF for combinations ("comb") and complete algorithms. The last two rows show the percentage of cells in the quasi-identifiers that are suppressed and the entropy change from the baseline as a percentage.

**Table 10:** Main results for clinical file

	TOTAL_LOS_DAYS						TOTAL_LOS_WEEKS					
	**0.2**		**0.05**		**0.04**		**0.2**		**0.05**		**0.04**	

	**Comb**.	**Complete**	**Comb**.	**Complete**	**Comb**.	**Complete**	**Comb**.	**Complete**	**Comb**.	**Complete**	**Comb**.	**Complete**

**GENDER_CODE**	0.002	0.003	0.005	0.005	0.006	0.006	0.002	0.004	0.004	0.01	0.004	0.012

**AGE_GROUP**	2.5	6.2	7.6	16.7	8.86	19.2	1.32	3.7	4.2	9.8	4.8	11.25

**TOTAL_LOS_XXX**	1.2	6.8	1.8	12.3	1.9	13.3	1.2	4.7	1.8	7.08	1.9	7.4

**MRDx**	15.8	16.4	30.4	29	33	31.1	10.2	10.8	19.8	20	21.6	21.7

**CMG_CODE**	15.8	16.4	30.4	29	33	31.1	10.2	10.8	19.8	20	21.6	21.7

**DIAG_BLOCK**	11.4	5.4	24.2	10.2	27	11.1	6.96	3.5	15.1	6.8	16.5	7.5

**DIAG_CHAPTER**	2.22	0.14	5	0.28	5.6	0.34	1.4	0.066	3.3	0.16	3.7	0.19

**CCI_CODE**	7.4	9.16	12.3	14	13.2	14.8	4.9	6.7	8.9	11.22	9.6	12

**SHORT_CCI**	7.4	9.16	12.3	14	13.2	14.8	4.9	6.7	8.9	11.22	9.6	12

**Total % Cells Suppressed**	**7.08**	**7.74**	**13.77**	**13.94**	**15.1**	**15.1**	**4.6**	**5.2**	**9.1**	**9.6**	**9.92**	**10.4**

**Entropy (%)**	**100**	**123**	**201**	**237**	**219**	**259**	**50**	**68**	**114**	**143**	**126**	**158**

Missingness (as a percentage of all records) for different probability threshold levels and levels of LOS detail for the quasi-identifiers in the second PUMF for combinations ("comb") and complete algorithms. The last two rows show the percentage of cells in the quasi-identifiers that are suppressed and the entropy change from the baseline as a percentage.

Two versions of the geographic PUMF file were created, one at each of the two levels of geography (PROV_ALL and PROV_REGION). PROV_REGION treats the country as consisting of three regions and the territories. Also, two versions of the clinical PUMF file were created, one that shows the individual days of LOS for the first week and one that treats 1 to 7 total days of stay as a single category (LOS_DAYS and LOS_WEEKS). This distinction is important because the vast majority of stays are less than one week. The multiple versions of the PUMF file allow us to consider the impact of these alternative generalizations on information loss.

The results for both suppression algorithms, combinations and complete, are also shown in Table 9 and Table 10. For each data set and threshold we show the percentage of values within each quasi-identifier that are suppressed, and the percentage of all cells across all quasi-identifiers that are suppressed. The entropy as a percentage change from a baseline (indicated by 100%) is given for each column.

The missingness and entropy results indicate that: (a) there is less missingness and less information loss with the 0.05 threshold than for the 0.04 threshold, (b) the combinations suppression algorithm produces less information loss overall than the complete suppression algorithm, and (c) the PROV_REGION and TOTAL_LOS_WEEKS data sets have less information loss overall. For the latter finding, this reflects that the generalizations for the PROV_REGION and TOTAL_LOS_WEEKS data sets were offset by the significant reduction in suppression, giving them a higher utility.

Based on these results, and to maintain data utility the authors and CIHI decided to use the 0.05 threshold for creating a PUMF, and use the PROV_ REGION and TOTAL_LOS_WEEKS data sets with the combinations suppression algorithm.

### Distribution of Missingness

The second set of results show how the missingness is distributed in the data. We consider how missingness on the diagnosis code variable distributed by province (Table 11), age group (Table 12), and length of stay (Table 13) for the PUMF files for the diagnosis variable. These results are only presented here for the PROV_REGION and TOTAL_LOS_WEEKS PUMF files at the 0.05 probability threshold.

**Table 11:** The percentage of missingness on CMG_CODE by region

Province	CMG_CODE (% Missing)
East (10.8%)	30.3

Central (45.5%)	19.1

West (43.2%)	16.6

Territories (0.3%)	53.0

The percentage in parentheses next to each region is its percentage of the total file. The probability threshold level was 0.05.

**Table 12:** The percentage of missingness on CMG_CODE by age group for each PUMF

	CMG_CODE (% Missing)	
**AGE_GROUP**	**PUMF 1**	**PUMF 2**

0-4	5.2	5.3

5-9	23	13.8

10-14	28.6	19

15-19	20.4	15.6

20-24	14.8	11

25-29	11.2	8.7

30-34	12	9.3

35-39	17.9	13.6

40-44	26	20.3

45-49	25.7	22.1

50-54	25.9	23

55-59	25.8	23.7

60-64	23.9	23.6

65-69	23.4	24.4

70-74	22.8	24.4

75-79	20.4	24.6

80-84	19.4	24.9

85-89	19.2	23.3

90+	18	21.1

The probability threshold level was 0.05.

**Table 13:** The percentage of missingness on CMG_CODE by total LOS in weeks for the second PUMF

TOTAL_LOS_WEEKS (days)	CMG_CODE (% Missing)
1-7	11

8-14	44

15-21	60

22-28	68

29-35	75

36-42	81

43-49	83

50-56	79

57-63	78

64-70	100

71-77	100

78-84	100

176+	80

The probability threshold level was 0.05.

### Performance of Suppression Algorithms

**Figure 8:**
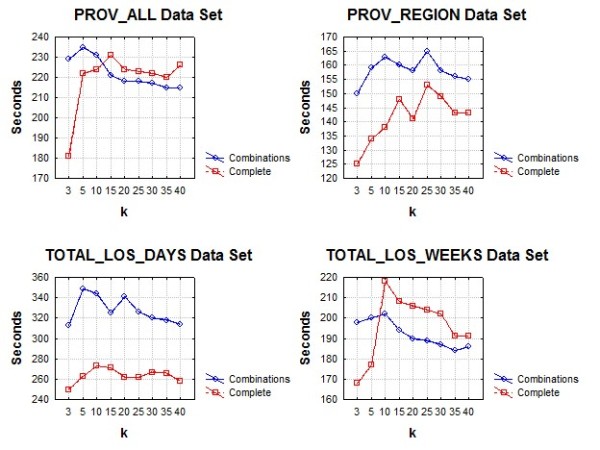
**Time in seconds to perform the suppression using each of the two algorithms on the four resultant data sets as the k value is changed**.

The first set of results are shown in Figure 8, which show overall performance in seconds. This varies from 2 minutes to six minutes on the full DAD population data set. There are a number of trends that can be observed:

• Because the PROV_ALL and TOTAL_LOS_DAYS data sets have variables with more response categories, they will take longer to suppress. This is expected because that means they also have a larger number of equivalence classes that the algorithm has to iterate through and hence apply more suppression.

• The differences between the two algorithms tend to be more marked at lower values of *k*, with the combinations algorithm taking longer. This is due to the complete algorithm reaching a solution quite fast at low values.

• One would expect that the combinations algorithm would take much more than the complete algorithm. Such a difference would be in phase 2 as both algorithms have the same phase 1. While there are differences, they are not dramatic. This is explained by the observation that earlier combinations result in suppressions that reduce the number of necessary iterations for subsequent combinations.

**Figure 9:**
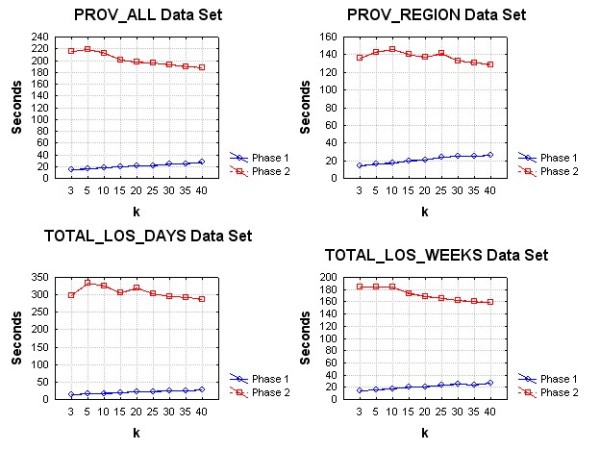
**Time to perform each of the phases of the combinations suppression algorithm for the four resultant data sets as the k value is changed**.

**Figure 10:**
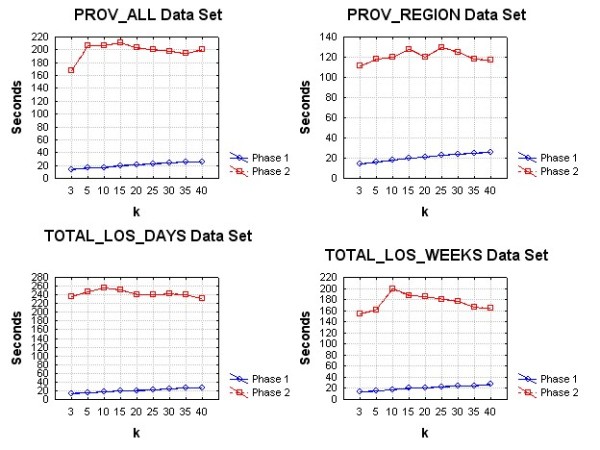
**Time to perform each of the phases of the complete suppression algorithm for the four resultant data sets as the k value is changed**.

The trend is for less time at low *k*, then rising, and eventually starting to fall again at the higher values of *k*. The explanation for the gradual reduction in computation time as *k *increases is that the suppression during phase 1 of both algorithms has more impact for the higher values of *k*, which results in fewer iterations for phase 2. This pattern is evident in the graphs of Figure 9 and Figure 10, where the phase 1 time increases with *k *reflecting more iterations, but phase 2 gradually decreasing.

The graphs in Figure 9 and Figure 10 show that phase 2 of the algorithm takes considerably more time. This is driven by the need to re-compute equivalence classes and their sizes at each iteration. Whereas phase 1 only considers individual quasi-identifiers.

For such large data sets the overall performance of the combinations algorithm was deemed acceptable, especially that the increment in time costs compared to the complete suppression algorithm was not significant.

**Figure 11:**
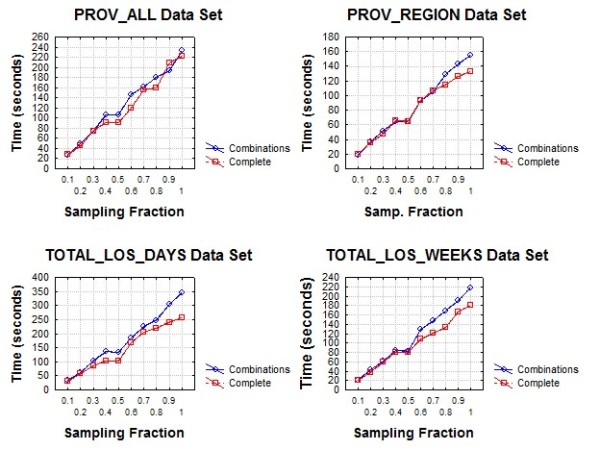
**Time in seconds to perform the suppression using each of the two algorithms on the four resultant data sets as the sampling fraction is changed**.

Figure 11 shows how the performance changes as the sampling fraction changes. In all cases the algorithms' time performance scales linearly with larger data sets. In general the gap in time between the combinations and complete algorithms is minimal at small sampling fractions, and widens as the sampling fraction increases.

## Discussion

### Summary

A substantial difference was found between the PROV_ALL and PROV_REGION data sets, and the TOTAL_LOS_DAYS and TOTAL_LOS_WEEKS data sets. The more generalized data sets tended to have considerably less information loss. Also, the clinical PUMF tended to have more suppression than the geographic PUMF because the LOS variable has more possible values on it (see Table 2). Therefore, we recommended using the PROV_REGION and TOTAL_LOS_WEEKS data sets as the basis for a PUMF.

There is an advantage to using the combinations suppression algorithm compared to the complete suppression algorithm. This is evident when we look at the percentage of total cells suppressed for the two algorithms and the difference in entropy.

Therefore, by using the 0.05 threshold, the more generalized versions of the two PUMF files, and the combinations algorithm we are able to produce a data set with significantly less information loss than would otherwise be the case. For example, for the geographic PUMF using PROV_ALL at the 0.04 threshold with the complete suppression algorithm results in 13.6% of the cells being suppressed, whereas our recommended PUMF has only 8.4% of its cells suppressed. For the second PUMF the differences are 15.1% versus 9.1%. In both cases there was a marked improvement in data utility as measured by the percentage of suppressed cells. Similarly, the complete suppression PROV_ALL data set at the 0.04 threshold had a 334% entropy value, compared to the combinations suppression PROV_REGION file with a 0.05 threshold which had less than two thirds of the entropy at 181%.

The variable with the most suppression was diagnosis code, with just under 20% of the records having suppression there. The least generalized data set with the 0.04 threshold and the complete suppression algorithm results in suppression closer to 30% on the diagnosis code variable.

The smallest provinces tend to have the most suppression: the Eastern region and the territories. Similarly, age groups that tend to have the most suppression are those with the fewest hospital visits (e.g., 5-14 years), and the age groups with the least suppression have the most hospital visits (e.g., newborns and toddlers in the 0-4 years age range). By far, the vast majority of stays are less than 7 days, and therefore this category has the least suppression. Longer stays are considerably less common, resulting in them having high levels of suppression.

Our combinations suppression algorithm results in less information loss than an algorithm that does not take combinations into account. It has acceptable performance on this large data set for k values up to 40, and scales linearly with the data set size.

### Practical Implications

One important reason for sampling was to ensure that the two PUMF files would not have the same individuals in them, precluding the possibility of linking the two files by an adversary. For example, using the same reasoning as in Additional file 3, any two independent samples with replacement from the population will have an expected number of records that can be matched correctly equal to *Kα *where *K *is the number of equivalence classes in the population and *α *is the sampling fraction. If we have 100,000 equivalence classes in the population and a 10% sampling fraction, then 10,000 records could be correctly matched on average. A higher sampling fraction would mean more records could be matched. In practice, an adversary would not know which 10,000 records were matched correctly, however, which may limit the meaningfulness of the matching exercise. Nevertheless, to err on the conservative side we wanted to ensure that the samples cannot overlap.

To contextualize our results with other disclosures of health information in Canada, the Statistics Canada census disclosure control process uses record level suppression, therefore the census PUMF does not have suppressed cells. It should also be noted that the census PUMF does not include any variables that have as many response categories as the DAD (e.g., the census only includes generalized occupation or industry codes with at most 25 possible categories, unlike diagnosis and intervention codes which are much more detailed). Therefore, in one sense it is not surprising that the amount of suppression for the DAD PUMF under the current specifications would be higher than we see in the census file. Furthermore, the Canadian census PUMF is still not generally available to the public, but has some restrictions on its disclosure. Another study which performed a re-identification risk assessment for a non-public disclosure of a pharmacy file noted that suppression on some variables of as much as 15% still provided utility to the end users [140]. This number is comparable to ours when one considers that the pharmacy file disclosure required a data sharing agreement and regular audits of the data recipients.

Rather than drawing the PUMF data from the suppressed population file, it may be better to remove the records with suppression on many quasi-identifiers or where key variables are suppressed, and then draw the PUMF files from this new population. That way there will be little or no cell suppression in the PUMF files. However, such a PUMF will be missing more records from patients living in small areas, with longer stays, and with rare diagnoses and interventions. It may be possible to compensate for such missingness by providing weights for the non-suppressed values.

Another approach to reduce missingness is to further generalize diagnosis and intervention codes. It is quite likely that further reductions in the number of categories on these variables would reduce missingness. However, it remains an empirical question whether additions of other levels to the current domain generalization hierarchies for these two variables will be meaningful.

The PUMF that we have described here may be of utility for many specific purposes, such as making it easier for the research community to confirm some published results, providing broader feedback to CIHI to improve data quality, training students and fellows, providing an easily accessible data set for researchers to prepare for analyses on the full DAD data set, and serving as a large data set for computer scientists and statisticians to evaluate analysis and data mining techniques. However, for complex research analysis, more detailed information would typically be needed than can be provided in a PUMF. The most appropriate action may be to produce an Analytical File that would be readily available to researchers and other users who satisfy some significant conditions regarding access and use of the data, including signing and being legally bound by data sharing agreements. Such a file would still need to have records with a low probability of re-identification, although the threshold used would likely be set higher, say 0.2, leading to reduced suppression and therefore greater utility of the released data. As noted earlier, higher thresholds are suitable when the data is being disclosed to trusted parties. Data sharing agreements establish the trust and so mitigate the risks from the higher threshold. The use of data sharing agreements is the approach used by the AHRQ in the US when disclosing state discharge abstract data for secondary purposes.

### Limitations

The analysis presented here does not address protection against attribute disclosure. In a final PUMF that would be released to the public, protections against attribute disclosure would normally need to be implemented as an added precaution.

## Conclusions

The public availability of detailed hospital discharge abstract data could benefit many communities, including health and data analysis researchers, educators, students, as well as the data custodians themselves. However, the public disclosure of health information can only be done if it is credibly de-identified. In this paper we have described how Canadian discharge abstract data can be de-identified, and clarified the costs in terms of data quality of doing so. Our de-identification utilized new metrics, methods and algorithms to maximize the utility of the data and to provide strong privacy guarantees. Furthermore, the process we followed highlighted the tradeoffs that need to be considered by a data custodian when making data available. But challenges remain for the disclosure of detailed health information, for example, the generalization of diagnosis codes to reduce the number of unique codes but retaining sufficient detail is an area for future work.

## Abbreviations

DAD: Discharge abstact database; PUMF: Public use microdata file; AHRQ: Agency for Healthcare Research and Quality; PRAM: Post randomization method; Prec: Precision; DM: Discernability metric; MD: Minimal distortion; CM: Classification metric; IG: Information gain; PL: Privacy loss.

## Competing interests

The authors declare that they have no competing interests.

## Authors' contributions

KEE designed the study, performed the data analysis, developed the metrics, developed the suppression algorithm, and contributed to writing the paper. DP designed the study, performed the data analysis, and contributed to writing the paper. FD developed the metrics and contributed to the complexity analysis. GK contributed to the literature review. All of the authors have read and approved the final manuscript.

## Pre-publication history

The pre-publication history for this paper can be accessed here:

http://www.biomedcentral.com/1472-6947/11/53/prepub

## Supplementary Material

Additional file 1 **Appendix A: Generalizing Diagnosis and Intervention Codes**. How the original diagnosis and intervention codes were generalized. Click here for file

Additional file 2 **Appendix B: Constructing Identification Databases from Public Sources**. A summary of public and semi-public registries in Canada and how these can be combined to re-identify certain types of individuals. Click here for file

Additional file 3 **Appendix C: Measuring the Probability of Re-identification from Matching Data Sets**. The derivation and simulation results for the re-identification metrics that are used in the risk assessment in the paper. Click here for file
