# Supplementary material for: De-identifying a public use microdata file from the Canadian national discharge abstract database
Source: BMC Med Inform Decis Mak. 2011 Aug 23;11:53. doi: 10.1186/1472-6947-11-53 (PMC3179438; doi:10.1186/1472-6947-11-53)
Supplement: Additional file 1 — Appendix A: Generalizing Diagnosis and Intervention Codes. How the original diagnosis and intervention codes were generalized. [file 1472-6947-11-53-S1.PDF]

Appendix A: Generalizing Diagnosis and Intervention Codes

In this appendix we describe how the different levels of precision for the diagnosis and intervention codes have been derived.

1.1 Diagnosis Codes

ICD-10-CA is the Canadian enhancement to ICD-10. There were 8967 different most responsible diagnoses represented in the subset of the DAD we used.

There is a hierarchy within the coding structure, with six levels. These are chapter, block, sub-block, category, sub-category, and Canadian enhancement.

A chapter is the highest level in the coding scheme. There are 21 usual chapters plus two for special purposes. In general the first character of an ICD-10-CA code identifies the chapter.

Blocks are the next highest level. There were 195 diagnosis blocks represented in our file. CMGs (Case Mix Groups) are not exclusively created by aggregating diagnoses. These groups are designed to aggregate acute care in-patients with similar clinical and resource utilization characteristics. The CMG grouping algorithm involves a hierarchy. First, patients are assigned to a Major Clinical Category (MCC) based on their most responsible diagnosis (MRDx). They are then assigned to Diagnosis and Intervention Partitions based on the interventions and/or diagnoses present. In the Diagnosis partition the final grouping is diagnosis-driven, while it is intervention-driven in the Intervention partition.

1.2 Intervention Codes

The DAD uses Canadian Classification of Health Intervention (CCI) codes. There were 8767 of these codes in our file. The hierarchy of intervention codes is not as clear as that for diagnoses. Interventions are grouped into Sections, which are consistently designated by the first character of the code. Sections 1, 2, and 3 (Physical/Physiological Therapeutic Interventions, Diagnostic Interventions, and Diagnostic Imaging interventions) have similar internal code structures, with the second character generally indicating a body system or region, and the third character further refining the location. The fourth and fifth characters identify what was done, while the sixth and seventh (and eighth and ninth) identify the technique used in more detail. This pattern is not maintained for the other Sections (Obstetrical and Fetal Interventions; Cognitive, Psychosocial and Sensory Diagnostic and Therapeutic Interventions; Other Healthcare Interventions; Therapeutic Interventions Strengthening the Immune System and/or Genetic Composition).

To generalize the interventions the approach taken was to reduce the detail both on the location and the technique. To accomplish this, the third, sixth and subsequent characters were suppressed. This generalization works fairly well as a prototype, producing 872 codes for principal intervention. It is however simplistic. Within Sections 1, 2, and 3, some systems use more than one letter for the second character. Within Section 6 the third character is always the same. Within Section 7 the second character is always the same. Within Section 8 the second and third characters aren't hierarchical. Within Section 5 this generalization appears to be reasonable. In Sections 1, 2, and 3, all the two-character combinations for a system have been recoded together. In Section 8 both the second and third characters were suppressed. In Section 7 the third character was not suppressed.
