# Supplementary material for: De-identifying a public use microdata file from the Canadian national discharge abstract database
Source: BMC Med Inform Decis Mak. 2011 Aug 23;11:53. doi: 10.1186/1472-6947-11-53 (PMC3179438; doi:10.1186/1472-6947-11-53)
Supplement: Additional file 2 — Appendix B: Constructing Identification Databases from Public Sources. A summary of public and semi-public registries in Canada and how these can be combined to re-identify certain types of individuals. [file 1472-6947-11-53-S2.PDF]

Appendix B: Constructing Identification Databases from Public Sources

In the main body of the paper we described the use of population registries in re-identification attacks. However, the term “population registries” is a simplification because in practice it is not possible to legally obtain a population registry in Canada for the purposes of re-identification. The term used in the disclosure control literature is “identification databases” which contain the quasi-identifiers as well as full identity information. These identification databases can cover the whole popluation, or more likely some subset of the population.

Previous research has suggested that it is relatively easy to construct identification databases about certain sub-populations in Canada because of the information available about them in public and semi-public registries [1, 2]. Three specific sub-populations which have been determined to be risky are: homeowners, those in professions with professional associations that publish comprehensive membership lists (e.g., physicians and lawyers), and civil servants. We will therefore only consider these three sub-populations as being those at risk of re-identification by linking to public registries.

This appendix describes the process for constructing an identification databases for the three at-risk member groups: homeowners, professionals whose professional associations publish membership lists, and civil servants. The objective is to illustrate how this can be done in practice.

It should be noted that our focus is the use of Canadian public registries since this is the context of our study. Although the general process would be similar when creating identification databases in other jurisdictions, such as the US, the specific public and semi-public registries would differ.

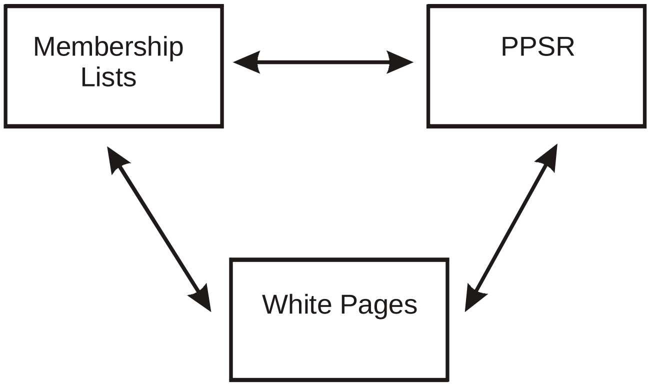

Figure 1: The steps for linking public and semi-public data sources to construct a identification database for professionals.

1.1.1 Professionals

In a previous study we described the process for the construction of an identification database for physicians and lawyers in Ontario using publicly available registries [1]. The results were that date of birth was determined for 40% of physicians and 45% of lawyers, gender for 100% of both, and home address for 60% of physicians and 45% of lawyers.

The general process consists of taking the publicly available lists of Ontario professionals (from their professional associations), and then search the publicly available Private Property Security Registry (PPSR) of Ontario. The PPSR contains address information as well as dates of birth. For those individuals that have a record in the PPSR, we would get their basic demographics. Some of the details (such as address) can be verified by cross-checking against other osurces such as the White Pages, and Google Maps (to confirm that the address is a residence and not, say, a factory).

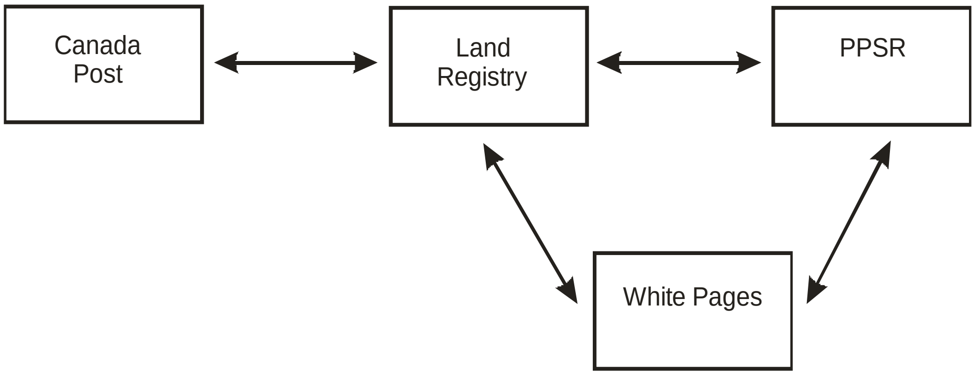

Figure 2: The four main public source databases used to construct an identification database for a home owner sub-population. The PPSR is the Property Security Registry of Ontario. Also, as described below, along with Canada Post's Postal Code Directory, Google Maps may be used to search for postal codes.

1.1.2 Homeowners

We constructed an identification database for the Forward Sortation area (FSA) "N3E" in Ontario. This particular FSA was chosen because it has a relatively small number of dwellings and had mixed use properties (i.e., residential and commercial). We believe it is reasonable to say that a similar process can be followed for any other FSA in Canada.

The first step was to get a list of all the properties within that FSA from Canada Post. Canada Post has a reverse lookup function on its website that can be used to retrieve a listing of all street addresses in a given postal code. Satellite imagery is available for many parts of the country – particularly urban areas – through several web sources (e.g., Google Maps). By using such sources, it can be determined whether a given postal code or address is industrial, commercial, mixed use, or primarily residential in nature.

The next step was to search each property contained in the particular postal code in the Land Registry to get a comprehensive list of the names of present and past homeowners for each property. The telephone directory was then used to confirm this information.

The last step was then to search the PPSR of Ontario. Figure 2 shows the overall linking done to create the homeowner identification database.

This resulted in a list of names and addresses for 70 homeowners. For these 70 homeowners, we obtained the gender and date of birth for 10 of them (14%). The ages ranged from 44 to 64, with 8 male and 2 female homeowners.

In a second study we selected two suburban residential postal codes, one in Ottawa and one in Toronto, and attempted to create an identification database for their homeowners. There were 15 houses in the Ottawa postal code and 20 houses in the Toronto postal code. Using a process similar to the one described above, we were able to obtain the date of birth for 33% of the Ottawa homeowners, and 40% of the Toronto ones. Gender could be derived for 87% and 95% respectively.

1.1.3 Civil Servants

The total Canadian federal civil service has approximately 386,630 employees. The Government Electronic Directory Services (GEDS) is an on-line database of a subset of the federal civil service. GEDS has more than 170,000 records [3], which is less than 50% of all civil servants. There are a number of reasons for this: federal government organizations choose whether or not to list their employees, employees in some cases have to take specific action to be listed in GEDS, some employees are exempted because of the nature of their work, and employees in federal government organizations involved in law enforcement, defense and the secret service would not be listed.

GEDS is therefore not a comprehensive listing of federal employees. However, within the scope of GEDS, we selected a sample of 40 employees working in Ontario-based federal healthcare related departments and attempted to obtain their name, gender, date of birth, and address from the PPSR and the telephone directory as described earlier.

We were able to obtain the home address for 50% of the selected sample (20/40), home telephone number for 40% (16/40), gender for 100% (40/40), and date of birth for 22.5% (9/40) of the GEDS entries that we selected. A complete record with name, address, date of birth, and gender (all of the quasi-identifiers) was obtained for 22.5% (9/40) of the sample.

1.2 References

1. El Emam K, Jabbouri S, Sams S, Drouet Y, Power M. *Evaluating common de-identification heuristics for personal health information.* Journal of Medical Internet Research, 2006; 8(4):e28.

2. El Emam K, Jonker E, Sams S, Neri E, Neisa A, Gao T, Chowdhury S. *De-Identification Guidelines for Personal Health Information*. 2007; Report produced for the Office of the Privacy Commissioner of Canada: Ottawa.

3. *GEDS FAQ*. 2005 [Available from: http://direct.srv.gc.ca/cgi-bin/direct500/TE?FN=faq.htm.
