# Supplementary material for: De-identifying a public use microdata file from the Canadian national discharge abstract database
Source: BMC Med Inform Decis Mak. 2011 Aug 23;11:53. doi: 10.1186/1472-6947-11-53 (PMC3179438; doi:10.1186/1472-6947-11-53)
Supplement: Additional file 3 — Appendix C: Measuring the Probability of Re-identification from Matching Data Sets. The derivation and simulation results for the re-identification metrics that are used in the risk assessment in the paper. [file 1472-6947-11-53-S3.PDF]

Appendix C: Measuring the Probability of Re-identification from Matching Data Sets

In this appendix we derive re-identification metrics for two very specific attacks. We refer to Figure 1 for an illustration of the data sets involved in both attacks.

Population Database

	QUASI-IDENTIFIERS		
ID	Prov	Gender	Year of Birth
1	ON	Male	1959
2	ON	Male	1962
3	ON	Female	1955
4	ON	Male	1959
5	ON	Female	1942
6	ON	Female	1975
7	ON	Female	1966
8	ON	Female	1987
9	ON	Male	1975
10	ON	Male	1978
11	ON	Female	1964
12	ON	Male	1959
13	ON	Female	1975
14	ON	Male	1967
15	ON	Male	1961
16	ON	Male	1974
17	ON	Female	1978
18	ON	Female	1961
19	ON	Female	1968
20	ON	Male	1973
21	ON	Female	1966
22	ON	Male	1967
23	ON	Male	1971
24	ON	Female	1974
25	ON	Female	1977
26	ON	Male	1968
27	ON	Male	1954
28	ON	Female	1952
29	ON	Female	1956
30	ON	Female	1988
31	ON	Female	1941

Sample

PUMF (D)

	QUASI-IDENTIFIERS		
ID	Prov	Gender	Year of Birth
1	ON	Male	1959
3	ON	Female	1955
4	ON	Male	1959
7	ON	Female	1966
8	ON	Female	1987
9	ON	Male	1975
13	ON	Female	1975
14	ON	Male	1967
15	ON	Male	1961
16	ON	Male	1974
17	ON	Female	1978
18	ON	Female	1961
23	ON	Male	1971
24	ON	Female	1974
25	ON	Female	1977
26	ON	Male	1968

Matching

Sample

Population Registry (I)

	IDENTIFYING VARIABLE	QUASI-IDENTIFIERS		
ID	Name	Prov	Gender	Year of Birth
3	Alice Brown	ON	Female	1955
4	Hercules Green	ON	Male	1959
5	Alicia Freds	ON	Female	1942
9	Bill Nash	ON	Male	1975
10	Albert Blackwell	ON	Male	1978
11	Beverly McCulsky	ON	Female	1964
21	Kathy Last	ON	Female	1966
22	Deitmar Plank	ON	Male	1967
27	Almond Zipf	ON	Male	1954
28	Alex Long	ON	Female	1952
29	Britney Goldman	ON	Female	1956
30	Lisa Marie	ON	Female	1988
31	Natasha Markhov	ON	Female	1941

Figure 1: Matching the PUMF against a population registry. The ID field is only included here to make it easier to see which records are included in each data set. In reality there would be no consistent ID field available across the data sets.

The adversary has the PUMF (data set D) and a population registry (data set I). Let D and I be two simple random samples from a population (say, represented by the census). Note that D and I must have common records but that I is not necessarily a subset of D or vice versa, in other words, there is a possible overlap between the two for any matching to be successful: $D \cap I \neq \varnothing$.

Assume that the set of population equivalence classes is denoted by Q, and $j \in Q$. We denote C_j as the number of records in a population equivalence class. Let the set of equivalence classes in the PUMF be denoted by J where $J \subseteq Q$, and f_j is the size of an equivalence class j in the PUMF and F_j is the size of an equivalence class in the population registry.

If an adversary attempts to match records in the two data sets, then it is necessary that there are some records in common between the two data sets. Say the adversary is trying to match records in equivalence class j, let m_j be the number of records common to both the PUMF and the population registry and $m_j \geq 0$.

1 Re-identification Attack 1

Under this attack, the adversary selects a *single* random record from the PUMF and is attempting to match that single record to the population registry. The question is what is the probability of a correct match ?

1.1 Derivation

We let $\boldsymbol{f}_j$ be the set of records in equivalence class j in the PUMF, and $\boldsymbol{F}_j$ the set of records in equivalence class j in the population registry. Now, assume that an adversary selects a record for patient r from the population registry, and tries to re-identify that patient in the PUMF. Also, let $r \in \boldsymbol{f}_j$, then the probability of correctly re-identifying patient r is denoted by $\Pr\left(d\left(r\right)\right)$ and is calculated as follows:

$$\begin{aligned}\Pr\left(d\left(r\right)\right) &= \Pr\left(d\left(r\right) \wedge \left(r \in \boldsymbol{F}_j \vee r \notin \boldsymbol{F}_j\right)\right) \\ &= \Pr\left(d\left(r\right) \wedge r \in \boldsymbol{F}_j\right) + \Pr\left(d\left(r\right) \wedge r \notin \boldsymbol{F}_j\right) \\ &= \Pr\left(d\left(r\right) \wedge r \in \boldsymbol{F}_j\right) + 0 \\ &= \Pr\left(r \in \boldsymbol{F}_j\right)\Pr\left(d\left(r\right) \middle| r \in \boldsymbol{F}_j\right) \\ &= \frac{m_j}{f_j}\frac{1}{F_j}\end{aligned}$$

But what is the value of m_j ?

To get the expected value of m_j recall that D and I are drawn from the same population. Let $\boldsymbol{f}_j$ and $\boldsymbol{F}_j$ be the set of records in equivalence class j in D and I respectively, (note that $f_j \leq C_j$ and $F_j \leq C_j$ and that $\boldsymbol{f}_j$, $\boldsymbol{F}_j$ or both might be empty), we need to calculate:

- The probability that $m_j = x$ for all x in $1,...,\min\left(f_j, F_j\right)$:

$$P(m_j = x) = \frac{\binom{C_j - F_j}{f_j - x}\binom{F_j}{x}}{\binom{C_j}{f_j}}$$

- The expected value of m_j would then be:

$$
\begin{aligned}
E(m_j) &= \sum_{x=1}^{\min(f_j, F_j)} xP(m_j = x) \\
&= \sum_{x=1}^{\min(f_j, F_j)} x \frac{\binom{C_j - F_j}{f_j - x}\binom{F_j}{x}}{\binom{C_j}{f_j}} \\
&= \frac{F_j}{C_j} f_j
\end{aligned}
$$

Hence the expected disclosure risk for a record *r* becomes:

$$
\Pr\left(d\left(r\right)\right) = \frac{m_j}{f_j}\frac{1}{F_j} = \frac{\frac{F_j}{C_j} f_j}{f_j}\frac{1}{F_j} = \frac{1}{C_j}
$$

This means the actual probability of re-identification is equal to the reciprocal of the size of the equivalence class in the overall population (which can be represented by the census). This probability is not dependent on the size of the identification database nor on the size of the PUMF.

1.2 Simulation

We performed a simulation to demonstrate the accuracy of our derivation. The simulation is that of a matching experiment based on the scenario described under attack 1.

There are a number of different matching approaches that can be used for this kind of simulation. Some authors have used probabilistic and distance-based matching methods in re-identification attack experiments [1, 2]. In this simulation we use exact matching methods for a number of reasons:

- Our theoretical derivation of the probability above assumes exact matching. Given that our objective is to validate the theoretical derivation, it is appropriate to use the same matching approach.
- There has been a dearth of work on re-identification risk metrics assuming non-exact matching by an adversary, and therefore exact matching during an attack is a common assumption.
- In our simulation we do not distort the data sets to introduce errors. This removes one of the advantages of using probabilistic and distance-based matching techniques.
- Exact matching will provide a worse case probability of re-identification because it assumes ideal conditions. Therefore, it gives us a conservative evaluation of risk.

We assumed that the size of the population equivalence class, the C_j value, varies as: 10, 100, 500, or 1000. We then drew randomly 10,000 times an artificial PUMF equivalence class and a population registry equivalence class. It does not matter what the value on the quasi-identifiers in this equivalence class are or how many quasi-identifiers there are since they would all have the same value. The sampling fraction for the PUMF was denoted by s_1 and for the population registry by s_2. A record r was drawn randomly from the PUMF and matched to the population registry. The proportion of matches that were correct was computed.

In most situations the sampling fractions for the PUMF and the registry from the whole population are going to be low. We therefore varied the sampling fractions from 0.1 to 0.5 in increments of 0.1.

The results are shown in the tables below. As can be seen, the simulated values are quite close to the theoretical value under the different simulated study points, suggesting that the equations above provide reasonably good estimates of the risk.

s_2 \ s_1	**0.1**	**0.2**	**0.3**	**0.4**	**0.5**
**0.1**	0.0015	0.0015	0.001	0.001	0.001
**0.2**	0.0009	0.001	0.0011	0.0007	0.0008
**0.3**	0.001	0.0003	0.001	0.0011	0.001
**0.4**	0.0008	0.001	0.0009	0.0009	0.0008
**0.5**	0.0002	0.0005	0.0011	0.0012	0.0009

Table 1: Simulation of matching experiment with population equivalence class equal to 1,000. The theoretical expected value is 0.001.

s_2 \ s_1	**0.1**	**0.2**	**0.3**	**0.4**	**0.5**
**0.1**	0.0023	0.00215	0.00195	0.0016	0.00195
**0.2**	0.00165	0.00155	0.00215	0.0018	0.0022
**0.3**	0.00315	0.00185	0.00215	0.00245	0.00175
**0.4**	0.00155	0.0021	0.002	0.0014	0.0018
**0.5**	0.00215	0.0027	0.0015	0.00155	0.0022

Table 2: Simulation of matching experiment with population equivalence class equal to 500. The theoretical expected value is 0.002.

		s_1				
		**0.1**	**0.2**	**0.3**	**0.4**	**0.5**
	**0.1**	0.011	0.0136	0.012	0.0093	0.0106
	**0.2**	0.0197	0.0106	0.0095	0.0056	0.00815
s_2	**0.3**	0.0162	0.008	0.008	0.009	0.011
	**0.4**	0.0054	0.01	0.009	0.01	0.0099
	**0.5**	0.0091	0.0079	0.01	0.01	0.0095

Table 3: Simulation of matching experiment with population equivalence class equal to 100. The theoretical expected value is 0.01.

		s_1				
		**0.1**	**0.2**	**0.3**	**0.4**	**0.5**
	**0.1**	0.114	0.104	0.101	0.115	0.094
	**0.2**	0.123	0.127	0..127	0.247	0.099
s_2	**0.3**	0.094	0.0997	0.06	0.06	0.149
	**0.4**	0.104	0.124	0.124	0.123	0.098
	**0.5**	0.1	0.2	0.147	0.1	0.157

Table 4: Simulation of matching experiment with population equivalence class equal to 10. The theoretical expected value is 0.1.

2 Re-identification Attack 2

For this re-identification attack, the adversary is attempting to match *all* of the records in the PUMF to the population registry or vice versa. The question is how many records can be matched successfully ?

2.1 Derivation

An adversary tries to match the two databases one equivalence class at a time. In other words, for every equivalence class j in the PUMF the adversary matches the records in $\boldsymbol{f}_j$ to the records in $\boldsymbol{F}_j$. Lacking any additional information apart from the matching quasi-identifiers, the adversary can match any two records from the two corresponding equivalence classes at random with equal probability. We assume that the adversary uses one-to-one mappings (i.e., no two records in $\boldsymbol{f}_j$ can be mapped to the same record in $\boldsymbol{F}_j$).

Assume that h records in $\boldsymbol{f}_j$ have been matched to h different records in $\boldsymbol{F}_j$ for some $h \in \{1,..., f_j - 1\}$, then the adversary draws randomly an $h+1^{\text{th}}$ record from $\boldsymbol{f}_j$, say r, and tries to match it with its corresponding record, if any, in $\boldsymbol{F}_j$. Then the probability that the $h+1^{\text{th}}$ record r will be correctly matched to its corresponding record, say R (which might or might not belong to $\boldsymbol{F}_j$), can be calculated as follows (note that, $P(r \in \mathbf{m}_j) = P(R \in \mathbf{F}_j)$, where $\mathbf{m}$ is the set of overlapping records):

$P_r = P(\ r \in \boldsymbol{m}_j)\ P(\ R$ is not yet matched$|\,R \in \boldsymbol{F}_j)\ P(\ r$ is matched to $R\,|\ R$ was not matched and $R \in \boldsymbol{F}_j)$

$$= \frac{m_j}{f_j} \frac{\binom{F_j - 1}{h}}{\binom{F_j}{h}} \frac{1}{F_j - h} = \frac{m_j}{f_j} \frac{F_j - h}{F_j} \frac{1}{F_j - h} = \frac{m_j}{f_j F_j}$$

Hence, the expected number of records that would be disclosed from equivalence class $\boldsymbol{f}_j$ is:

$$\sum_{j \in \langle \boldsymbol{f}_j, \boldsymbol{F}_j \rangle} \frac{m_j}{f_j F_j} = \frac{m_j \min\left(f_j, F_j\right)}{F_j f_j} \qquad \text{.............................. (1)}$$

where $\langle \boldsymbol{f}_j, \boldsymbol{F}_j \rangle$ returns the smaller set.

And the total expected number of records that would be correctly matched in the PUMF is:

$$\sum_{j \in Q} \frac{m_j \min\left(f_j, F_j\right)}{F_j f_j} \qquad \text{.............................. (2)}$$

But what is the value of m_j? This is computed exactly as for attack 1 above such that $E\left(m_j\right) = \frac{F_j}{C_j} f_j$.

Hence the total expected number of records that would be correctly matched in the PUMF becomes (from equation 2):

$$\sum_{j \in Q} \frac{F_j}{C_j} f_j \frac{\min\left(f_j, F_j\right)}{F_j f_j} = \sum_{j \in Q} \frac{\min\left(f_j, F_j\right)}{C_j} \qquad \text{.............................. (3)}$$

2.2 Simulation

We performed a simulation to determine how well our analytical derivation above matches actual results. Given that in practice sampling fractions from a population are going to be small, we simulated a matching experiment

with varying sampling fractions for the PUMF (s_2) and the population registry (s_1) from 0.1 to 0.5 in 0.1 increments.

We used actual data sets to represent the population. The first data set is the fatal crash information database from the department of transportation with 101,034 observations [3]. The quasi-identifiers used were age, gender, race, and date of death. The second data set is the hospital state inpatient database for the state of Washington for 2007 available from AHRQ. This data set has 227,500 records, with the following quasi-identifiers: year of birth, gender, length of hospital stay, and ZIP code.

We computed the number of matching records 10,000 times for each combination of s_1 and s_2. For each of these iterations the difference between the simulation number of matching records and the expected number as calculated from equation (3) above was computed. The average difference across all iterations was calculated, and presented as a proportion of all records in the PUMF. Therefore, the results are the average error presented as a proportion of the PUMF. If this number is negative it means that the derivation in equation (3) underestimates the actual expected value, and if it is positive then the derivation overestimates the expected value.

The results of the simulations in Table 5 and Table 6 show that the average error is generally quite small. As the population registry grows in size the error tends to increase. The derivation slightly underestimates the expected value as our results are consistently negative.

		s_1				
		**0.1**	**0.2**	**0.3**	**0.4**	**0.5**
	**0.1**	-0.001421	-0.006362	-0.013792	-0.023664	-0.035588
	**0.2**	-0.001226	-0.004614	-0.009827	-0.017870	-0.026737
s_2	**0.3**	-0.001037	-0.003553	-0.007563	-0.013600	-0.021253
	**0.4**	-0.000545	-0.002716	-0.005667	-0.010287	-0.016667
	**0.5**	-0.000569	-0.001909	-0.004617	-0.007818	-0.012460

Table 5: Simulation of the error in the number of records matched divided by the size of the PUMF for the fatal crash information system data set.

s_2 \ s_1	0.1	0.2	0.3	0.4	0.5
**0.1**	-0.007543	-0.027706	-0.054856	-0.065120	-0.082568
**0.2**	-0.002278	-0.0098442	-0.023248	-0.040385	-0.059095
**0.3**	-0.000955	-0.0042554	-0.010876	-0.020803	-0.033342
**0.4**	-0.000434	-0.0020005	-0.005183	-0.010511	-0.018599
**0.5**	-0.0002131	-0.0010426	-0.002665	-0.005388	-0.010042

Table 6: Simulation of the error in the number of records matched divided by the size of the PUMF for the Washington state inpatient data set.

3 References

1. Torra V, Domingo-Ferrer J. Record linkage methods for multidatabase data mining, in *Information Fusion in Data Mining*. 2003. p. 101-132.
2. Torra V, Abowd J, Domingo-Ferrer J. Using Mahalanobis distance-based record linkage for dislcosure risk assessment. 2006; Springer LNCS. p. 233-242.
3. Department of Transportation. Fatal crash information. Available from: [http://www-fars.nhtsa.dot.gov/main.cfm]
